# Artificial Intelligence‐Assisted Urine Cytology for Noninvasive Detection of Muscle‐Invasive Urothelial Carcinoma: A Multi‐Center Diagnostic Study with Prospective Validation

**DOI:** 10.1002/advs.202508977

**Published:** 2025-07-25

**Authors:** Runnan Shen, Fan Jiang, Xiaowei Huang, Guibin Hong, Yun Luo, Huan Wan, Ye Xie, Mengyi Zhu, Yun Wang, Bohao Liu, Ping Qin, Yahui Wang, Haoxuan Wang, Hongkun Yang, Zhen Lin, Rui Chen, Nengtai Ouyang, Jian Huang, Tianxin Lin, Shaoxu Wu

**Affiliations:** ^1^ Department of Urology Sun Yat‐sen Memorial Hospital of Sun Yat‐sen University Guangzhou 510120 China; ^2^ Guangdong Provincial Key Laboratory of Malignant Tumour Epigenetics and Gene Regulation Guangdong‐Hong Kong Joint Laboratory for RNA Medicine Sun Yat‐sen Memorial Hospital of Sun Yat‐sen University Guangzhou 510120 China; ^3^ Guangdong Provincial Clinical Research Centre for Urological Diseases Guangzhou 510120 China; ^4^ CellsVision Medical Technology Services Co., Ltd. Guangzhou 510260 China; ^5^ Department of Urology The Third Affiliated Hospital of Sun Yat‐sen University Guangzhou 510630 China; ^6^ Department of Pathology Sun Yat‐sen Memorial Hospital of Sun Yat‐sen University Guangzhou 510120 China; ^7^ Department of Pathology The Third Affiliated Hospital of Guangzhou Medical University Guangzhou 510150 China; ^8^ Department of Urology The Shen‐Shan Central Hospital Shanwei 516600 China

**Keywords:** artificial intelligence, muscle invasive, prospective validation, urine cytology, urothelial carcinoma

## Abstract

Accurate preoperative diagnosis of muscle invasion (MI) is critical for urothelial carcinoma (UC) management. The aim is to evaluate whether artificial intelligence (AI) model based on urine cytology can accurately detect MIUC and compare its performance with radiologist assessments. UC patients underwent liquid‐based urine cytology from four centers are included for model development/validation. Performance of the precision urine cytology AI solution for MI (PUCAS‐M) is validated across multicenter cohorts and compared to radiologists’ assessments (including CT/MR, MR accounted for 40.7%). Clinical utility is assessed for initial diagnosis, recurrence detection, and neoadjuvant therapy. PUCAS‐M achieves an area under the receiver operation curve (AUROC) of 0.857 (95% CI: 0.820‐0.895) in the whole validation cohort, which is significantly higher (*P*‐value = 0.005) than radiologists (0.773, 95% CI: 0.727–0.818). The integration of radiologists' diagnosis and PUCAS‐M (mPUCAS‐M) significantly increases the sensitivity of radiologists from 63.9% to 83.3% in bladder cancer and from 76.9% to 90.3% in upper‐tract UC. Lastly, in the neoadjuvant therapy subgroups, mPUCAS‐M maintains an improved AUROC (ranging from 0.857‐0.865), whereas radiologist assessments' performance decline. PUCAS‐M provides accurate, non‐invasive MI detection method, particularly valuable for equivocal imaging. Integration with clinical data enhances diagnostic precision, offering a scalable solution for UC management.

## Graphical abstract

Accurate preoperative detection of muscle‐invasive urothelial carcinoma (MIUC) is crucial for clinical decision‐making. This study developed PUCAS‐M, an artificial intelligence (AI) model that analyzes urine cytology to assist in differentiating MIUC from non‐MIUC. In multicenter validation, both PUCAS‐M and multi‐modal PUCAS‐M exhibited robust diagnostic accuracy. The model consistently maintained high performance across diverse clinical scenarios, highlighting its potential as a valuable tool in clinical practice.

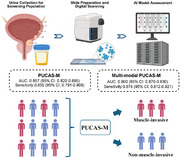

## 1 Introduction

Urothelial carcinomas (UC) represent a major type of cancer affecting the urinary system and rank as the fourth most prevalent tumor type.^[^ ^1^, ^2^, ^3^ ^]^ It can arise in either the lower urinary tract, which includes the bladder and urethra, or in the upper urinary tract, such as the renal pelvis and ureter. Bladder cancer (BC) constitutes approximately 90–95% of all UC cases. In contrast, upper tract UC (UTUC) are relatively rare, comprising only 5–10% of cases.^[^ ^4^ ^]^ Muscle invasion (MI) and the corresponding T stage are the most critical prognostic factors for UC and play a decisive role in informing treatment decisions, especially for BC.^[^ ^2^ ^]^ According to the MI status, BC can be divided into non‐muscle‐invasive BC (NMIBC) and muscle‐invasive BC (MIBC), each exhibiting distinct treatment protocols. For NMIBC, transurethral resection of bladder tumor (TURBT) remains the gold‐standard method, followed by bacillus Calmette‐Guerin (BCG) or adjuvant chemotherapy.^[^ ^1^ ^]^ For MIBC, treatment typically involves neoadjuvant chemotherapy with or without immunotherapy, followed by radical cystectomy and pelvic lymph node dissection (LND).^[^ ^5^, ^6^ ^]^ For UTUC, MI staging (≥ pT2) is observed in 60% of cases, with 5‐year cancer‐specific mortality rates ranging between 21% and 59%.^[^ ^7^, ^8^ ^]^ While complete resection of the kidney and ureter is considered the standard treatment, the decision to perform LND is highly contingent upon the pre‐operation assessment of MI status^[^ ^4^ ^]^ Recent studies have described that neoadjuvant chemotherapy can be safely delivered to achieve pathologic downstaging and improved survival in MI UTUC.^[^ ^9^ ^]^ Thus, accurate MI staging holds significant implications for treatment planning. However, traditional diagnostic strategies still face several limitations.

The most accurate MI staging method for BC prior to cystectomy is primarily based on TURBT and histopathological reports of resected specimens. However, earlier studies have demonstrated that histopathological results may present discrepancies between clinical and pathological staging due to variations in resection techniques.^[^ ^10^, ^11^ ^]^ Unlike BC, UTUC cannot be assessed for MI through diagnostic resection. In addition, ureteroscopy tumor biopsies underestimate the pathological stage of UTUC in up to 46% of cases.^[^ ^12^ ^]^ Notably, these methods are invasive, costly, and require anesthesia. Computed tomography (CT) and magnetic resonance (MR) are commonly used and recommended for the staging of UC. In discriminating MIBC and NMIBC, MRI showed superior diagnostic accuracy compared to CT owing to its favorable soft‐tissue resolution.^[^ ^13^ ^]^ However, previous studies have documented that the diagnostic accuracy of MRI remains suboptimal.^[^ ^14^, ^15^ ^]^ With the introduction of the Vesical Imaging‐Reporting and Data System (VI‐RADS) scoring system, the sensitivity and specificity of MRI can reach up to 92% and 87%, respectively.^[^ ^16^, ^17^ ^]^ Nevertheless, in areas with insufficient medical resources or centers lacking specialized training, MRI reports often fail to provide a definitive diagnosis, which usually results in missed diagnoses among referred patients. Regarding UTUC, CT urography is regarded as the most accurate diagnostic method, superior to MR urography. However, given that UTUC patients are generally at a higher risk of renal failure, the potential risks associated with the injection of iodine contrast agents should be paid attention to. Thus, there is a pressing need to develop novel, non‐invasive, and less harmful tools to assist in the detection of MI in UC.

Urine cytology, the most convenient and cost‐effective urine test method, plays a crucial role in the diagnosis of UC.^[^ ^18^ ^]^ Nevertheless, it is limited by low sensitivity and the potential inability to capture atypical or malignant cells.^[^ ^19^, ^20^ ^]^ Our previous study developed and validated an artificial intelligence (AI)‐based model termed the precision urine cytology AI solution (PUCAS) to enhance the sensitivity of urine cytology.^[^ ^21^ ^]^ As anticipated, PUCAS could identify imaging features associated with UC and establish corresponding links at the cytological and pathological levels.^[^ ^22^ ^]^ Furthermore, it enhanced the sensitivity of urine cytology, ranging from 24.2% to 49.0%, reduced the rate of misdiagnoses of low‐grade and early‐stage UC, and showed promise as an alternative to fluorescence in situ hybridization (FISH). In the traditional diagnostic process, urine cytology cannot be used to discriminate the MI stage of UC. However, we hypothesize that urine cytology images contain information on MI, which is not observable by the naked eye, given that a previous study reported that CSP7 status in FISH was an independent factor for predicting MIBC.^[^ ^23^ ^]^

In the present study, the design framework of the previously constructed PUCAS model was extended to develop a model for detecting MI in UC (PUCAS‐M) in a large, multi‐center observation cohort. Three clinical scenarios, including initial diagnosis, recurrence detection, and neoadjuvant treatment, were established to evaluate its clinical utility and robustness. Prospective validation and comparison to radiologist assessments (including CT or MR assessments) were conducted to investigate the ability of PUCAS‐M to assist in clinical decision‐making in real‐world settings. Finally, a multi‐modal model (mPUCAS‐M) was also developed to investigate the degree of improvement over conventional procedures.

## 2 Experimental Section

### 2.1 Participants

**Figure 1:**
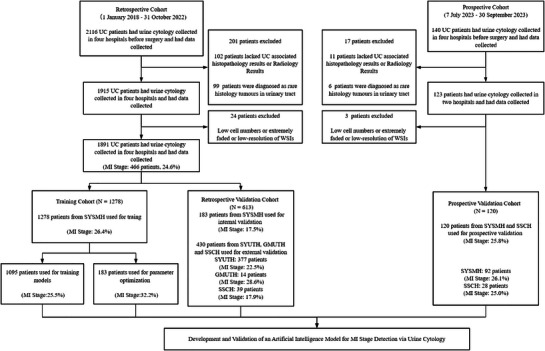
Flowchart of the study. WSIs, whole‐slide images; UC, urothelial carcinoma; MI, muscle invasion; SYSMH, Sun Yat‐sen Memorial Hospital of Sun Yat‐sen University; SYUTH, Third Affiliated Hospital of Sun Yat‐sen University; GMUTH, Third Affiliated Hospital of Guangzhou Medical University; SSCH, Central Hospital of Shen Shan.

In this multicenter observational study we included patients who underwent liquid‐based voided urine cytology examinations at four hospitals in China (**Figure** **1**). The retrospective cohort was collected from January 1, 2018 to October 31, 2022, from Sun Yat‐Sen Memorial Hospital of Sun Yat‐Sen University (SYSMH), the Third Affiliated Hospital of Sun Yat‐Sen University (SYUTH), the Third Affiliated Hospital of Guangzhou Medical University (GMUTH), and Shen‐Shan Central Hospital (SSCH). The prospective cohorts were collected from July 7, 2023, to September 15, 2023, at SYSMH and SSCH. The exclusion criteria were as follows: those who declined UC‐associated surgery and without histopathological or radiological (MR or CT) reports; or those who were pathologically confirmed non‐UC, given that the Paris System for Reporting Urinary Cytology (TPS) primarily detect UC.^[^ ^24^ ^]^ This prospective observational study was registered under ChiCTR2300073192. Data collection for the prospective trial was independently conducted and blinded to each other (Supporting Information).

This study was approved by the ethics committee of all participating hospitals. The requirements for informed consent for both retrospective and prospective studies were waived owing to the observational study design; however, each participant had provided written informed assent for the Collection and Application of Clinical Sample and Medical Data certified.

### 2.2 Procedures

All liquid‐based cytology slides were obtained from the archives of the pathology departments and stained using the Papanicolaou method (Supporting Information). Five types of scanners were adopted to generate whole‐slide images (WSIs) at 40 times magnification. Low‐quality slides, defined by low cell numbers (less than 20), extreme fading, or improper staining, were excluded from the analysis. The baseline characteristics of participants, including demographic characteristics and radiological and histopathological reports, were acquired from medical record archives. Data on race/ethnicity were not collected. The gold standard for MI detection was based on histopathological reports from transurethral resection of bladder tumor (TURBT), radical cystectomy, or radical nephroureterectomy. The radiological reports included CT and MR assessments. The MR criteria for BC were mainly based on VI‐RADS and we defined three MRI‐based diagnosis: a VI‐RADS score exceeding 3 was classified as muscle‐invasion, a score below 3 was classified non‐muscle‐invasion, and a score of 3 was classified as uncertainty. Two senior radiologists independently reviewed the radiological reports. Disagreements were adjudicated by a third expert (>20 years of experience). Patients from the SYSMH retrospective cohort were stratified at a 7:1 ratio and then assigned to a “training cohort” and an “internal validation cohort.” Similarly, patients from the training cohort were stratified into “model training” and “parameter optimization cohorts” at a 6:1 ratio. Patients from SYUTH, GMUTH, and SSCH in the retrospective and internal validation cohorts were assigned to “retrospective validation cohorts.” The whole prospective cohort was defined as the “prospective validation cohort.” Retrospective and prospective validation cohorts were combined to form a whole validation cohort for further subgroup analyses.

For model training, two expert cytopathologists (each with >15 years of experience in urine cytopathology) independently annotated urothelial cells at the single‐cell level, classifying them as normal, atypical, or malignant based on TPS criteria. Other cell types (e.g., glandular, squamous) were also annotated, while ambiguous or degenerated cells (including inflammation or chemotherapy associated change) were labeled as “others.” Representative examples are illustrated in Figure S1 (Supporting Information). A total of 315149 cells were annotated in the training cohort. For WSI‐level cytology diagnosis, a central consensus review was implemented to ensure objectivity. The primary diagnosis was determined by two cytopathologists. Disagreements were resolved by a third expert (>20 years of experience).

Building upon the established PUCAS framework, the PUCAS‐M system employed a three‐tiered analytical pipeline for MI detection. In the initial preprocessing phase, WSIs with a resolution of 50000×50000 pixels were systematically partitioned into non‐overlapping 1024×1024‐pixel patches at 0.25 µm per pixel resolution (40× magnification), ensuring computational efficiency while preserving critical cytological details. The subsequent feature extraction stage employed a multi‐model processing pipeline, where YOLOv7^[^ ^25^ ^]^ was used for the precise localization of atypical cells, EfficientNet^[^ ^26^ ^]^ was then applied to abstract cellular‐level features from the atypical cells identified by YOLOv7, whilst both RegNetY^[^ ^27^ ^]^ and ConvNeXt‐B^[^ ^28^ ^]^ were utilized for architectural pattern analysis. This combination of models collectively generated comprehensive patch‐level feature descriptors.

For whole‐slide interpretation, the system integrated an ensemble of attention‐based Bi‐LSTM^[^ ^29^ ^]^ networks for spatial context modeling, Transformer^[^ ^30^ ^]^ architectures for long‐range dependency analysis, and Top‐N Feature^[^ ^31^ ^]^ modules for discriminative feature selection. The final MI probability scores were derived through weighted averaging of all WSI‐level model outputs, maintaining the original training protocols, including optimized data augmentation strategies and class‐balanced cross‐entropy loss functions as detailed in our prior implementation.^[^ ^21^ ^]^ Details are summarized in Figures S2–S4 (Supporting Information).

To investigate the effect of PUCAS‐M on improving radiologists' diagnosis, MI status predictions from the urine cytology model, radiologist assessments' results (CT or MR), and epidemiological data (age, sex, cytology results, hematuria, smoking, and clinical scenarios) were integrated to construct multi‐modal PUCAS‐M (mPUCAS‐M). A LightGBM classifier, incorporating an oversampling strategy, was employed for model training and validation. To address the class imbalance caused by the varying incidence of MI status, positive samples were oversampled to achieve a balanced distribution with negative samples. During model training using LightGBM, a grid search was carried out to optimize five hyperparameters: maximum depth, learning rate, number of estimators, minimum number of child samples, and the maximum number of leaves per decision tree. The optimal combination of hyperparameters, as determined by the grid search, was subsequently used to train the final model. Following model training, SHAP (SHapley Additive exPlanations) analysis was conducted to interpret the contribution of each feature, thereby enhancing model interpretability.

PUCAS‐M and mPUCAS‐M were initially validated in the retrospective and prospective validation cohorts. Furthermore, the retrospective and prospective validation cohorts were combined as a whole validation cohort for subgroup analyses. The diagnostic efficacy of the models was evaluated across diverse clinical subgroups, which were defined by factors such as cytology results, age, sex, hematuria, and different clinical scenarios, including initial diagnosis, recurrence detection, and neoadjuvant treatment. To assess the practical use of PUCAS‐M and mPUCAS‐M, their diagnostic performance was evaluated for BC and UTUC and compared with that of radiologist assessments (including comparison with CT/MR separately). Finally, the models were validated in the radiology Tx group to determine their utility in clinical decision‐making.

### 2.3 Outcomes

Model performance was principally evaluated using the area under the receiver operating curve (AUROC). Other performance measures, encompassing sensitivity, specificity, accuracy, positive predictive value (PPV), and negative predictive value (NPV), were secondary endpoints.

### 2.4 Statistical Analysis

The data were presented as *n* (%) for categorical variables and median (interquartile range, IQR) for continuous variables. Intergroup comparisons were performed using the Chi‐square test for categorical data and Kruskal‐Wallis test for continuous variables. Statistical analyses and data visualization were conducted using R software (version 4.2.0) and Prism 9 (GraphPad Software). Receiver operating characteristic (ROC) curves, AUROC, accuracy, sensitivity, specificity, and decision clinical analysis were employed to assess model performance (pROC package, epiR package, rmda package). The Delong test was used to compare two ROCs. The Pearson's χ2 test was utilized to assess discrepancies in sensitivity and specificity between various diagnostic methods. Net reclassification improvement (NRI) and integrated discrimination improvement (IDI) were used to assess the improved predictive ability of models compared to those of radiologist assessments. All analyses were prespecified. Two‐sided *P* values less than 0.050 were considered statistically significant.

### 2.5 Ethics Approval Statement

This study was approved by the ethics committee of all participating hospitals (SYSKY‐2023‐521‐01).

### 2.6 Patient Consent Statement

The requirements for informed consent for both retrospective and prospective studies were waived because of the observational design; however, each participant had provided written informed assent for the Collection and Application of Clinical Sample and Medical Data certified and approved by the ethics committee of all participating hospitals.

### 2.7 Clinical Trial Registration

The approved registration number is ChiCTR2300073192.

## 3 Results

A total of 2116 eligible UC patients were included in the retrospective cohort study, with data collected from January 1, 2018, to October 31, 2022. Additionally, 140 participants were enrolled in the prospective study between July 7, 2023, and September 15, 2023. Based on the predetermined exclusion criteria, 225 cases (10.6%) from the retrospective group and 20 cases (14.3%) from the prospective group were excluded from the final analysis.

A total of 1891 urine cytology images from the retrospective cohort were utilized to develop and validate PUCAS‐M. Images from SYSMH (1461 images, 77.3%) were divided into a training set (1278 images, 67.6%) and an internal validation set (183 images, 9.7%). The training set was further stratified into a model development subset (1095 images, 57.9%) and a parameter optimization subset (183 images, 9.7%). For external validation, images from SYUTH (377 images, 19.9%), GMUTH (14 images, 0.7%), and SSCH (39 images, 2.1%) were combined into a single external validation cohort (430 images, 22.7%). In the prospective cohort, 120 urine cytology images were included for validation, comprising images from SYSMH (92 images, 76.7%) and SSCH (28 images, 23.3%) (Figure 1). The clinical characteristics of the training and validation cohorts are detailed in **Table** **1**. The MR assessments counted for 40.7% in the whole validation cohorts. No missing data were observed across any cohorts.

**Table 1:** Baseline characteristics of tumor patients in the training and validation cohorts (Data are *n* (%) or median (IQR). SYSMH, Sun Yat‐sen Memorial Hospital of Sun Yat‐sen University; SYUTH, The Third Affiliated Hospital of Sun Yat‐sen University; GMUTH, The Third Affiliated Hospital of Guangzhou Medical University; SSCH, The Central Hospital of Shen‐Shan; UTUC, upper tract urothelial carcinoma; PUNLMP, papillary urothelial neoplasms of low malignant potential; CIS, carcinoma in situ; HGUC, high‐grade urothelial carcinoma; SHGUC, SHGUC, suspicious for high‐grade urothelial carcinoma; AUC, atypical urothelial cells; NHGUC, negative for high‐grade urothelial carcinoma; CT, computed tomography; MR, magnetic resonance).

	Retrospective cohort					Prospective cohort
	SYSMH training cohort (*N* = 1278)	SYSMH validation cohort (*N* = 183)	SYUTH validation cohort (*N* = 377)	GMUTH validation cohort (*N* = 14)	SSCH validation cohort (*N* = 39)	Prospective validation cohort (*N* = 120)
**Age**	65.00 [57.00, 72.00]	64.00 [58.00, 73.00]	67.00 [59.00, 75.00]	70.50 [63.50, 78.50]	68.00 [62.50, 74.50]	65.00 [59.00, 73.25]
**Gender**						
Male	1015 (79.4%)	149 (81.4%)	286 (75.9%)	12 (85.7%)	32 (82.1%)	95 (79.2%)
Female	263 (20.6%)	34 (18.6%)	91 (24.1%)	2 (14.3%)	7 (17.9%)	25 (20.8%)
**Haematuria**						
Yes	821 (64.2%)	123 (67.2%)	250 (66.3%)	11 (78.6%)	31 (79.5%)	85 (70.8%)
No	457 (35.8%)	60 (32.8%)	127 (33.7%)	3 (21.4%)	8 (20.5%)	35 (29.2%)
**Smoking**						
Yes	336 (26.3%)	46 (25.1%)	127 (33.7%)	4 (28.6%)	18 (46.2%)	34 (28.3%)
No	942 (73.7%)	137 (74.9%)	250 (66.3%)	10 (71.4%)	21 (53.8%)	86 (71.7%)
**Clinical scenario**						
Routine screening	891 (69.7%)	119 (65.0%)	326 (86.5%)	10 (71.4%)	32 (82.1%)	78 (65.0%)
Recurrence detection	387 (30.3%)	64 (35.0%)	51 (13.5%)	4 (28.6%)	7 (17.9%)	42 (35.0%)
Neoadjuvant treatment	146 (11.4%)	23 (12.6%)	22 (5.8%)	0 (0.0%)	4 (10.3%)	18 (15.0%)
**UC type**						
BC	973 (76.1%)	148 (80.9%)	241 (63.9%)	13 (92.9%)	28 (71.8%)	85 (70.8%)
UTUC	305 (23.9%)	35 (19.1%)	136 (36.1%)	1 (7.1%)	11 (28.2%)	35 (29.2%)
**Cancer grade**						
PUNLMP	972 (76.1%)	132 (72.1%)	203 (53.8%)	10 (71.4%)	18 (46.2%)	87 (72.5%)
Low‐grade	191 (14.9%)	33 (18.0%)	146 (38.7%)	3 (21.4%)	8 (20.5%)	24 (20.0%)
High‐grade	115 (9.0%)	18 (9.8%)	28 (7.4%)	1 (7.1%)	13 (33.3%)	9 (7.5%)
**Cancer stage**						
Ta	477 (37.3%)	80 (43.7%)	175 (46.4%)	5 (35.7%)	22 (56.4%)	46 (38.3%)
CIS	33 (2.6%)	3 (1.6%)	7 (1.9%)	1 (7.1%)	0 (0.0%)	3 (2.5%)
T1	430 (33.6%)	68 (37.2%)	110 (29.2%)	4 (28.6%)	10 (25.6%)	40 (33.3%)
T2	175 (13.7%)	10 (5.5%)	41 (10.9%)	3 (21.4%)	5 (12.8%)	20 (16.7%)
T3	116 (9.1%)	17 (9.3%)	32 (8.5%)	0 (0.0%)	2 (5.1%)	8 (6.7%)
T4	47 (3.7%)	5 (2.7%)	12 (3.2%)	1 (7.1%)	0 (0.0%)	3 (2.5%)
**Cytology results**						
NHGUC	305 (23.9%)	49 (26.8%)	107 (28.4%)	2 (14.3%)	13 (33.3%)	32 (26.7%)
AUC	363 (28.4%)	47 (25.7%)	125 (33.2%)	2 (14.3%)	13 (33.3%)	27 (22.5%)
SHGUC	118 (9.2%)	11 (6.0%)	54 (14.3%)	7 (50.0%)	3 (7.7%)	9 (7.5%)
HGUC	492 (38.5%)	76 (41.5%)	91 (24.1%)	3 (21.4%)	10 (25.6%)	52 (43.3%)
**Radiology assessments**						
CT	549 (43.0%)	78 (42.6%)	271 (71.9%)	6 (42.9%)	18 (46.2%)	61 (50.8%)
MR	729 (57.0%)	105 (57.4%)	106 (28.1%)	8 (57.1%)	21 (53.8%)	59 (49.2%)

**Figure 2:**
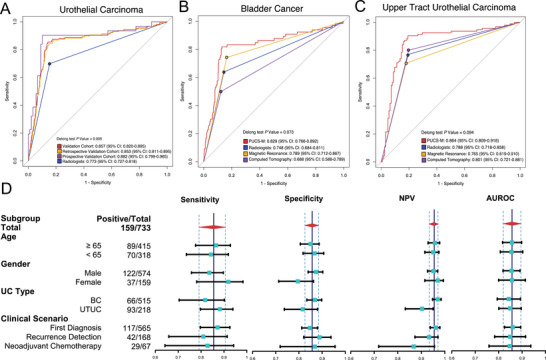
Performance of PUCAS‐M in validation cohorts. A) ROC curves of PUCAS‐M in detecting MIUC in validation cohort and different clinical scenarios, and compared with radiologist assessments. B) ROC curve of PUCAS‐M in detecting MIBC in validation cohorts and compared with radiologist assessments (including CT and MR separately). C) ROC curve of PUCAS‐M in detecting MI UTUC in validation cohorts and compared with radiologist assessments (including CT and MR separately). D) Corresponding sensitivities, specificities, NPVs, and AUROCs in different subgroups of validation cohorts, classified by age, gender, UC type, and clinical scenarios. PUCAS‐M, the precision urine cytology AI solution for muscle‐invasive; UC, urothelial carcinoma; ROC, receiver operating characteristic; MIUC, muscle invasive urothelial carcinoma; MIBC, muscle invasive bladder cancer; BC, bladder cancer; UTUC, upper tract urothelial carcinoma; NPV, negative predictive value; AUROC, area under the receiver operating characteristic; CI, confidence interval; AI, artificial intelligence; CT, computed tomography; MR, magnetic resonance.

PUCAS‐M achieved an AUROC of 0.857 (95% CI: 0.820–0.895) in the whole validation cohort, which was significantly higher (*P* value = 0.005) than that of radiologist assessments (0.773, 95% CI: 0.727–0.818). When stratified by time, PUCAS‐M also showed favorable discriminatory performance in the retrospective (0.853, 95% CI: 0.811‐0.895) and prospective validation cohorts (0.882, 95% CI: 0.799‐0.965) (**Figure** **2**A). In BC, PUCAS‐M showed a higher diagnostic performance (0.829, 95% CI: 0.766‐0.892) compared to radiologist assessments (0.748, 95% CI: 0.684‐0.811). MR showed a better discrimination ability (0.789, 95% CI: 0.712‐0.867) than CT but didn't surpass PUCAS‐M (Figure 2B). In UTUC, PUCAS‐M effectively detected MI (0.864, 95% CI: 0.809‐0.918), and achieved a higher AUROC, outperforming radiologists (0.788, 95% CI: 0.718‐0.858). CT showed a better discrimination ability (0.801, 95% CI: 0.721‐0.881) than MR but also didn't surpass PUCAS‐M (Figure 2C). Besides, PUCAS‐M showed a sensitivity ranging from 0.844 (95% CI: 0.769‐0.902) to 0.903 (95% CI: 0.742‐0.980) and a specificity ranging from 0.849 (95% CI: 0.815‐0.880) to 0.899 (95% CI: 0.817‐0.953) across the retrospective and prospective validation cohorts (Figure 2D, Table S1, Supporting Information). It also demonstrated an improved sensitivity and AUROC for the detection of MI compared to radiologists in both BC and UTUC patients. The results showed significance when compared to CT in BC (Table S2, Supporting Information). Meanwhile, PUCAS‐M showed robust and consistent performance in subgroup analyses based on age, sex, and different clinical scenarios. In the neoadjuvant treatment subgroup, PUCAS‐M showed a sensitivity of 0.828 (95% CI: 0.642‐0.942) and specificity of 0.868 (95% CI: 0.719, 0.956) (Figure 2D, Table S1, Supporting Information). The sensitivity and AUROC of PUCAS‐M (sensitivity: 0.828, 95% CI: 0.642, 0.942; AUROC: 0.789, 95% CI: 0.661, 0.916) were higher than those of radiologist assessments (sensitivity: 0.643, 95% CI: 0.441, 0.814; AUROC: 0.607, 95% CI: 0.484, 0.730) especially CT (sensitivity: 0.455, 95% CI: 0.167, 0.766; AUROC: 0.523, 95% CI: 0.323, 0.722) (Table S2, Supporting Information). In radiology Tx cases, PUCAS‐M achieved a high sensitivity of 0.935 (95% CI: 0.821, 0.986) in UC, 0.800 (95% CI: 0.284, 0.995) in BC, and 0.951 (95% CI: 0.835, 0.994) in UTUC, thereby enabling the model to identify more MI patients for further radiological evaluation (Table S3, Supporting Information).

To interpret the performance of PUCAS‐M, subgroup analyses were performed across three distinct cytology result categories: negative for high‐grade urothelial carcinoma (NHGUC), atypical urothelial cells (AUC), and high‐grade urothelial carcinoma (HGUC, including suspicious for high‐grade urothelial carcinoma [SHGUC]), considering the substantial heterogeneity in visual morphological assessment. As anticipated, PUCAS‐M demonstrated relatively superior discriminatory ability in NHGUC and HGUC (AUROC > 0.85). In the AUC subgroup, which poses diagnostic challenges for urologists, PUCAS‐M still achieved satisfactory AUROC results (0.801, 95% CI: 0.707‐0.895) (Figure S5, Supporting Information). To evaluate the potential of urine cytology in predicting urothelial carcinoma T‐stage, heatmaps were generated to interpret the AI model's predictive outcomes. The AI‐identified high‐risk regions (primarily localized to nuclei, cytoplasm, and peri‐cellular background areas) demonstrated strong concordance with pathologist‐annotated suspicious cells (Figure S6, Supporting Information). This morphospatial profiling provides a visual reference for developing a non‐invasive T‐stage predictive model, supported by correlative radiological and histopathological findings (Figure S7, Supporting Information).

Moreover, given the robust diagnostic performance of the single‐modal PUCAS‐M, the value of mPUCAS‐M in enhancing diagnostic accuracy was examined. After integrating radiological results and clinical data, mPUCAS‐M showed a sensitivity ranging from 0.867 (95% CI: 0.796‐0.921) to 0.903 (95% CI: 0.742‐0.980) and a specificity ranging from 0.862 (95% CI: 0.828‐0.891) to 0.876 (95% CI: 0.790–0.937) across the retrospective and prospective validation cohorts (Table S4, Supporting Information). At the same time, mPUCAS‐M showed a higher sensitivity (0.874, 95% CI: 0.812‐0.921) and AUROC (0.900, 95% CI: 0.870‐0.930) compared to PUCAS‐M and radiologists (The results showed significance in both CT and MR) in validation cohorts (Table S5, Supporting Information). The NRI and IDI comparing radiologist assessments (CT/MR) and PUCAS‐M are presented in Table S6, Supporting Information). The performance of mPUCAS‐M in radiology Tx subgroup in validation cohorts is shown in Table S7 (Supporting Information). The DCA curves delineated that mPUCAS‐M offered higher clinical utility than radiologists and PUCAS‐M in the validation cohorts and subgroups (Figure S8, Supporting Information). For interpretation of PUCAS‐M, SHAP analysis was conducted to evaluate the contributions of different predictors on model outcomes. Interestingly, the results revealed that PUCAS‐M prediction, radiologists' assessments, and patient sex were the most significant predictors (Figure S9, Supporting Information).

**Figure 3:**
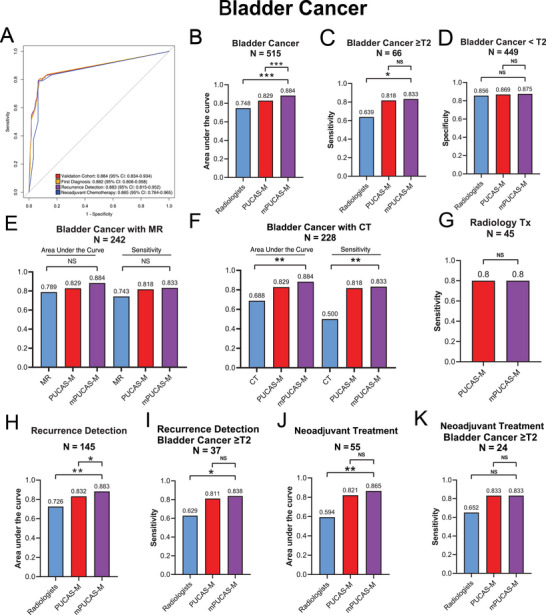
Performance of mPUCAS‐M in detecting MIBC in comparison with PUCAS‐M and radiologist assessments. A) ROC curves of mPUCAS‐M in detecting MIBC in validation cohorts and different clinical scenarios. B) Comparison of AUROCs of mPUCAS‐M, PUCAS‐M and radiologist assessments in detecting MIBC in validation cohort. C) Comparison of sensitivities of mPUCAS‐M, PUCAS‐M and radiologist assessments in detecting MIBC in validation cohort. D) Comparison of specificities of mPUCAS‐M, PUCAS‐M and radiologist assessments in detecting MIBC in validation cohort. (E) Comparison of AUROCs and sensitivities of mPUCAS‐M, PUCAS‐M and MR assessments in detecting MIBC in validation cohort. F) Comparison of AUROCs and sensitivities of mPUCAS‐M, PUCAS‐M and CT assessments in detecting MIBC in validation cohort. G) Comparison of sensitivities of mPUCAS‐M and PUCAS‐M in detecting MIBC in radiology Tx cohort. H,I) Comparison of AUROCs and sensitivities of mPUCAS‐M and PUCAS‐M in detecting MIBC in recurrence detection subgroup. J,K) Comparison of AUROCs and sensitivities of mPUCAS‐M and PUCAS‐M in detecting MIBC in neoadjuvant chemotherapy subgroup. The comparisons of sensitivity and specificity were assessed by Pearson's χ^2^ test. The comparison of AUROC was assessed by the DeLong test. *P < 0·05, **P < 0·01, ***P < 0·001. PUCAS‐M, The precision urine cytology AI solution for muscle‐invasive; mPUCAS‐M, the multi‐modal precision urine cytology AI solution for muscle‐invasive; ROC, receiver operating characteristic; MIBC, muscle invasive bladder cancer; AUROC, area under the receiver operating characteristic; CT, computed tomography; MR, magnetic resonance; NS, not significant; AI, artificial intelligence.

In BC, mPUCAS‐M achieved an AUROC of 0.884 (95% CI: 0.834‐0.934). Across the three clinical scenarios, the AUROCs ranged from 0·865 (95% CI: 0·764–0·905) to 0·883 (95% CI: 0·815–0·952) (**Figure** **3**A). In BC validation cohort, mPUCAS‐M showed a significantly higher AUROC (88.4%) compared to PUCAS‐M (82.9%) and radiologists (74.8%) (Figure 3B). Moreover, mPUCAS‐M showed a significantly higher sensitivity (83.3%) compared to radiologists (63.9%) (Figure 3C). Conversely, specificity was comparable between mPUCAS‐M, radiologist assessments, and PUCAS‐M (Figure 3D). mPUCAS‐M showed a higher but not significant AUROC and sensitivity than MR. But it showed significantly higher AUROC and sensitivity than CT (Figure 3E,F, Table S5, Supporting Information). In the hard‐to‐detect subgroup (radiology Tx), mPUCAS‐M displayed a sensitivity of 80.0% (Figure 3G, Table S7, Supporting Information). In the recurrence detection subgroup, mPUCAS‐M exhibited a significantly higher AUROC (88.3%) than radiologist assessments (72.6%) and PUCAS‐M (83.2%) (Figure 3H, Table S8, Supporting Information). Likewise, its sensitivity (83.8%) was significantly higher than radiologist assessments (62.9%) especially CT (Figure 3I, Table S8, Supporting Information). In the neoadjuvant treatment subgroup, mPUCAS‐M demonstrated outstanding discriminatory ability (86.5%) and diagnostic sensitivity (83.3%) for detecting MI after neoadjuvant therapy (Figure 3J,K, Table S8, Supporting Information). On the other hand, the AUROC of radiologist assessments remained low at 0.594, signifying that limited effectiveness in guiding bladder preservation strategies. Some patients who achieved down‐stage patients were still misclassified as manifesting MI. mPUCAS‐M seemed to overcome this shortcoming and can serve as a valuable diagnostic tool for assessing MI after neoadjuvant treatment in patients with MIBC. An illustrative case is presented in Figure S10A (Supporting Information), where mPUCAS‐M was able to accurately identify pathological downstaging in a BC patient following receipt of neoadjuvant chemotherapy and immunotherapy. A 70‐year‐old male cT3N1M0 MIBC patient received a planned treatment regimen consisting of four 21 d cycles of intravenous 200 mg tislelizumab on day 1, 1,000 mg m^−^^2^ gemcitabine on days 1 and 8, and 70 mg m^−^^2^ cisplatin on day 2. Prior to RC surgery, MR was conducted, and urine cytology was performed for mPUCAS‐M analyses. The MR results showed postoperative (TURBT) reactive changes and MI, whereas the postoperative (RC) pathology indicated a pathological stage of pT1. mPUCAS‐M predicted the absence of T2 disease, consistent with the final pathological report. Compared to radiologist assessments in assessing MIBC following neoadjuvant treatment, the NRI and IDI of mPUCAS‐M were nearly 0.5 and reached statistical significance (Figure S10B, Supporting Information). The baseline characteristics of treatment responder and non‐responder who received neoadjuvant treatment is shown in Figure S10C (Supporting Information).

**Figure 4:**
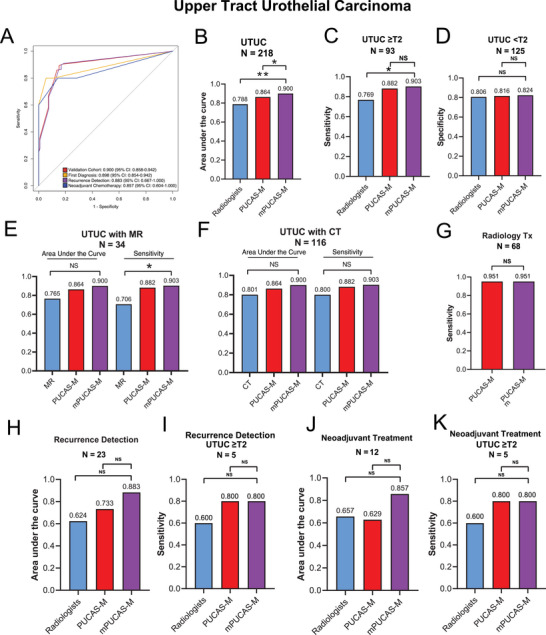
Performance of mPUCAS‐M in detecting MI UTUC in comparison with PUCAS‐M and radiologist assessments. A) ROC curves of mPUCAS‐M in detecting MI UTUC in validation cohorts and different clinical scenarios. B) Comparison of AUROCs of mPUCAS‐M, PUCAS‐M and radiologist assessments in detecting MI UTUC in validation cohort. C) Comparison of sensitivities of mPUCAS‐M, PUCAS‐M and radiologist assessments in detecting MI UTUC in validation cohort. D) Comparison of specificities of mPUCAS‐M, PUCAS‐M and radiologist assessments in detecting MI UTUC in validation cohort. E) Comparison of AUROCs and sensitivities of mPUCAS‐M, PUCAS‐M and MR assessments in detecting MI UTUC in validation cohort. F) Comparison of AUROCs and sensitivities of mPUCAS‐M, PUCAS‐M and CT assessments in detecting MI UTUC in validation cohort. G) Comparison of sensitivities of mPUCAS‐M and PUCAS‐M in detecting MI UTUC in radiology Tx cohort. H,I) Comparison of AUROCs and sensitivities of mPUCAS‐M and PUCAS‐M in detecting MI UTUC in recurrence detection subgroup. J,K) Comparison of AUROCs and sensitivities of mPUCAS‐M and PUCAS‐M in detecting MI UTUC in neoadjuvant chemotherapy subgroup. The comparison of sensitivity and specificity were assessed by Pearson's χ^2^ test. The comparison of AUROC was assessed by the DeLong test. *P < 0·05, **P < 0·01, ***P < 0·001. PUCAS‐M, The precision urine cytology AI solution for muscle‐invasive; mPUCAS‐M, the multi‐modal precision urine cytology AI solution for muscle‐invasive; ROC, receiver operating characteristic; AUROC, area under the receiver operating characteristic; CT, computed tomography; MR, magnetic resonance; AI, artificial intelligence; MI UTUC, muscle invasive upper tract urothelial carcinoma; NS, not significant.

In UTUC, the mPUCAS‐M model demonstrated excellent diagnostic performance, with an AUROC of 0.900 (95% CI: 0.858‐0.942). Specifically, the model achieved AUROCs ranging from 0.857 (95% CI: 0.604‐1.000) to 0.898 (95% CI: 0.854‐0.942) across the three distinct clinical scenarios for UTUC (**Figure** **4**A). Within the UTUC validation cohort, mPUCAS‐M exhibited significantly higher discriminatory ability (90.0%) compared to PUCAS‐M (86.4%) and radiologist assessments (78.8%) (Figure 4B), with a significantly higher sensitivity (90.3% vs 76.9% for radiologists; Figure 4C) while maintaining comparable specificity (Figure 4D). mPUCAS‐M showed a significantly higher sensitivity than MR. When compared to CT, it showed higher but not significant AUROC and sensitivity (Figure 4E,F, Table S5, Supporting Information). The multi‐modal approach demonstrated particular value in challenging UTUC cases, achieving a sensitivity of 95.1% in radiology Tx tumors (Figure 4G, Table S7, Supporting Information). For UTUC recurrence monitoring, mPUCAS‐M displayed high AUROC (Figure 4H, Table S8, Supporting Information) and sensitivity (Figure 4I, Table S8, Supporting Information) compared to the other two single‐modal models. In the neoadjuvant treatment subgroup for UTUC, mPUCAS‐M maintained strong discriminatory power (85.7%) and sensitivity (80.0%) for detecting MI post‐treatment, outperforming radiologists' AUROC of 65.7% and sensitivity of 60.0% (Figure 4J,K, Table S8, Supporting Information). A major advantage of mPUCAS‐M in UTUC lies in its high sensitivity and AUROC, addressing the challenge of accurately detecting MI before radical nephroureterectomy. Representative examples of mPUCAS‐M in accurately identifying MI UTUC are depicted in Figure S11 (Supporting Information).

## 4 Discussion

To provide a more non‐invasive, cost‐effective, and early diagnostic tool for evaluating the MI stage of UC, the PUCAS framework design was extended to develop the PUCAS‐M model, which was subsequently validated in a large cohort. Utilizing a multi‐stage framework and an integration decision‐making approach across multiple models, PUCAS‐M achieved an AUROC of 0.857 (95% CI: 0.820‐0.895) in the whole validation cohort, which was significantly higher than radiologist assessments (0.773, 95% CI: 0.727‐0.818). Moreover, PUCAS‐M demonstrated robust diagnostic performance in both retrospective and prospective validation cohorts, with sensitivity between 84.4% (95% CI: 76.9%‐90.2%) and 90.3% (95% CI: 74.2%‐98.0%) and specificity ranging from 84.9% (95% CI: 81.5%‐88.0%) to 89.9% (95% CI: 81.7%‐95.3%). The model exhibited particularly high sensitivity (93.5%, 95% CI: 82.1%‐98.6%) for the evaluation of radiologically indeterminate cases (Tx). Furthermore, the multi‐modal integration of radiologists' assessments with PUCAS‐M (mPUCAS‐M) yielded significant diagnostic improvements. In BC and UTUC cases, sensitivity increased from 63.9% to 83.3% and from 76.9% to 90.3%, respectively. Of note, mPUCAS‐M maintained stable diagnostic performance (AUROC: 0.857‐0.865) in the neoadjuvant therapy patient subgroups, where conventional radiological interpretation displayed insufficient performance.

Prognostic and therapeutic strategies for UC exhibit significant heterogeneity due to differences in MI status. MI UTUC (≥ pT2) indicates a poor prognosis, with 5‐year cancer‐specific mortality rates varying between 21% and 59%,^[^ ^8^ ^]^ whilst MIBC has a 5‐year overall survival rate <50% despite radical surgery.^[^ ^32^, ^33^ ^]^ Additionally, it carries a significant risk of progression to metastatic stages, at which point the 5‐year survival rate decreases to approximately 6%.^[^ ^24^ ^]^ Notably, recent advancements in research have demonstrated that neoadjuvant chemoimmunotherapy combination regimens for UC may achieve favorable tumor downstaging.^[^ ^6^, ^34^ ^]^ Thus, early diagnosis and timely therapeutic intervention are crucial for improving patients' quality of life, especially in cases where organ and renal function preservation becomes achievable. At present, the diagnosis of UC heavily relies on invasive pathological examinations. Particularly for UTUC, a complete resection is typically needed. Meanwhile, preoperative MRI or CT images are associated with suboptimal diagnostic accuracy.^[^ ^13^, ^35^ ^]^ Herein, among UC patients admitted over the past five years, including those transferred from other hospitals, the rate of indeterminate muscular layer invasion on imaging was as high as 15%. In UTUC patients, this rate reached up to 30%, partly ascribed to impaired renal function, precluding the use of contrast‐enhanced CT scans. Therefore, there is a pressing need to develop more non‐invasive and widely applicable diagnostic methods.

Urine testing has been proposed as an ideal candidate for this purpose, given that it directly interacts with tumor tissues, allowing for the non‐invasive collection of cellular samples.^[^ ^36^ ^]^ Moreover, its ease of collection, low psychological burden for patients, and suitability for large‐scale screening position it as a highly practical option. A previous study identified CSP7 status in FISH as an independent predictive factor for MIBC. Consequently, a cytogenetic‐clinical nomogram incorporating CSP7 status, radiology‐determined tumor size, and radiology‐determined clinical tumor stage was constructed, which yielded an AUROC of 0.743 (95% CI: 0.635 to 0.850).^[^ ^23^ ^]^ Similarly, Xiao et al. established a BCA‐specific DNA methylation signature from urine that could effectively discriminate between MIBC and NMIBC.^[^ ^37^ ^]^ Other novel biomarkers, including proteomics and microRNA, also exhibit promising potential for the identification of MIBC; nonetheless, their applications still require clinical validation.^[^ ^38^, ^39^ ^]^ These findings demonstrate that urine carries molecular signatures capable of identifying MI stages in UC patients. Despite urine cytology being the most widely used and cost‐effective clinical method, its diagnostic utility has been consistently limited by suboptimal sensitivity, particularly in early‐stage disease detection.^[^ ^40^ ^]^ Although the TPS has been extensively used to standardize diagnostic criteria, the misdiagnosis rate remains high, while the reporting rate of AUC generates significant diagnostic uncertainty for urologists.^[^ ^24^ ^]^ To date, research exploring the applicability of urine cytology in the diagnosis of MIUC remains scarce. Based on our previous research experience, we hypothesize that AI can identify features associated with muscle‐invasive UC in urine cytology images, thereby transforming it into a valuable tool for detecting muscle layer infiltration. Furthermore, the utilization of optimized algorithms, as well as the incorporation of a multi‐omics approach, significantly improved the diagnostic sensitivity of the model. More importantly, the results of this study are in line with and extend our prior research findings, forming an integrated system that not only enables large‐scale screening of UC patients but also guides further enhanced imaging or diagnostic biopsies. In cases where traditional diagnostic pathways yield inconclusive results, this AI‐based method may serve as a robust adjunct for clinical decision‐making.

Despite undergoing TURBT, patients with NMIBC exhibit a 1‐year recurrence rate of 40%,^[^ ^41^ ^]^ with over 20% of recurrent cases progressing to MIBC.^[^ ^42^ ^]^ According to another study, patients experiencing early recurrence are more likely to experience subsequent recurrences and have worse overall survival outcomes compared to those with late recurrence.^[^ ^43^ ^]^ Thus, regular monitoring is significant for the early detection of recurrence and progression in UC patients. Current guidelines recommend high‐risk tumor patients to undergo at least 15 endoscopic examinations over a 5‐year period. A secondary resection is also\ recommended for patients at the T1 stage and those with incompletely resected tumors, given that it can prolong recurrence‐free and progression‐free survival.^[^ ^44^, ^45^ ^]^ However, these procedures still carry significant risks. Particularly, bladder cancer patients who have undergone multiple TURBT surgeries are at a higher risk of bladder wall perforation due to inaccurate preoperative assessment. Thus, enhancing the predictive value for MIBC in recurrence detection or secondary resection holds considerable relevance. Our previous PUCAS model could accurately detect residual tumors with a high sensitivity of 96.6% and circumvent the need for unnecessary secondary TURBT with an NPV of 98.9%.^[^ ^21^ ^]^ Herein, the mPUCAS‐M model detected MIBC with a sensitivity of 0.833 (95% CI: 0.686, 0.930) and an NPV of 0.940 (95% CI: 0.881, 0.976) for recurrence detection, thereby offering more accurate surgical guidance.

In recent years, neoadjuvant chemotherapy and immunotherapy have demonstrated significant potential in the treatment of MIBC. The PUCAS‐M model can assist in screening patients eligible for neoadjuvant chemotherapy at the time of initial diagnosis. Nonetheless, monitoring the efficacy of neoadjuvant chemotherapy remains challenging.^[^ ^46^ ^]^ For instance, in patients exhibiting insensitivity to a gemcitabine + platinum regimen, timely switching to novel therapies such as immunotherapy should be considered.^[^ ^6^, ^46^ ^]^ In this regard, our clinical experience has unveiled that MRI carries the risk of misclassification, such as classifying a T1‐stage patient as T2+ (Figure S10, Supporting Information). Accurate tumor staging during and after treatment could potentially enable downstaging and bladder preservation for patients. Overall, the present study highlighted the outstanding performance of PUCAS‐M and mPUCAS‐M in evaluating the efficacy of neoadjuvant treatment, outperforming MRI. However, validation through larger clinical cohorts is still required.

Although UTUC represents a smaller subset of UC, it is associated with challenges in preoperative staging.^[^ ^4^ ^]^ Anatomically, unlike bladder cancer, UTUC cannot be evaluated for MI using transurethral resection techniques. And the preoperative T stage of UTUC is mainly based on radiology. Consequently, the integration of PUCAS‐M with conventional imaging modalities offers a more accurate diagnostic approach for UTUC (AUROC reached 0.900, 95% CI: 0.858‐0.942). In cases where contrast‐enhanced imaging is contraindicated, PUCAS‐M emerges as a valuable adjunctive screening tool, demonstrating high diagnostic efficacy (0.864, 95% CI: 0.809‐0.918). Muscle‐invasive UTUC has been linked to a poor prognosis, particularly following nephrectomy, where renal insufficiency can exacerbate the adverse effects of therapeutic agents.^[^ ^47^ ^]^ Neoadjuvant therapy in UTUC has shown promising results in tumor downstaging, with patients exhibiting superior tolerance to chemotherapy and an enhanced quality of life compared to those receiving postoperative adjuvant chemotherapy.^[^ ^9^ ^]^ Therefore, the precise preoperative identification of MI UTUC is crucial for neoadjuvant chemotherapy use. PUCAS‐M provides a refined tool for guiding therapeutic decision‐making in UTUC management.

The PUCAS‐M model demonstrated significant advantages in interpretability and automation through its innovative multilevel feature extraction framework. By employing a three‐model architecture that simulates expert consensus, the system comprehensively analyzes both patch‐level contextual features (including background contrast characteristics such as size, color, and brightness) and detailed cellular‐level features (encompassing hyperchromasia, coarse chromatin patterns, and irregular nuclear membranes). This dual‐level approach combines the high sensitivity of YOLOv7 for suspicious cell detection with the superior classification accuracy of RegNetY and ConvNext for abnormal field identification, with final diagnoses generated through an optimized weighted averaging algorithm. The workflow is fully automated; cytopathologists simply load slides into a scanner, which automatically digitizes and uploads WSIs to a cloud‐based system, where PUCAS‐M performs cell localization, feature analysis, and diagnostic scoring. This integrated solution not only enhances diagnostic accuracy by leveraging the strengths of complementary models but also significantly minimizes repetitive manual tasks, thereby optimizing workflow efficiency while maintaining clinical interpretability through its biologically relevant feature extraction approach.

Nonetheless, some limitations of this study merit acknowledgment. To begin, as an “end‐to‐end” AI‐based model, PUCAS‐M may lack transparency in its decision‐making process, which could hinder its acceptance among clinicians. Indeed, the “black‐box” nature of AI models often raises concerns about trust and reproducibility. Secondly, despite the promising performance of PUCAS‐M, its current deployment requires a labor‐intensive preprocessing workflow, including manual urine sample collection, slide preparation, digital scanning, quality processing, and further AI‐assisted diagnosis, resulting in a turnaround time of at least 1 day. Future efforts will focus on integrating automated systems, such as AI‐equipped toilets and cloud‐based platforms, to streamline the process and reduce diagnostic time. Another goal is to integrate the model with white‐light microscopy to achieve real‐time diagnosis. Moreover, PUCAS‐M was principally constructed using data derived from China centers, and external validation in other geographic settings is necessary. And the sample size for patients undergoing neoadjuvant chemotherapy or immunotherapy was limited, warranting further validation of PUCAS‐M to draw definitive conclusions. What's more, while the current mPUCAS‐M system demonstrates improved diagnostic performance through AI‐enhanced cytology, we acknowledge that further integration of novel urine‐based biomarkers (e.g., DNA methylation markers, protein signatures, or other molecular assays) may enhance its accuracy, particularly for detecting muscle invasion. Future studies should explore multimodal approaches combining cytology with such biomarkers to optimize diagnostic precision while maintaining clinical practicality.

In summary, PUCAS‐M is a robust, non‐invasive tool for MI detection in UC patients, which is applicable across various clinical scenarios. Its integration with clinical data further enhances diagnostic precision, offering a scalable solution to guide treatment strategies and reduce the need for invasive procedures.

## Conflict of Interest

The authors declare no conflict of interest.

## Supporting information



Supporting Information

## Data Availability

To protect patient privacy, image datasets and other patient‐related data are not publicly accessible, but all data are available upon reasonable request emailed to the corresponding author. To gain access, data requestors will need to sign a data‐access agreement.
