# Supplementary material for: Artificial Intelligence‐Assisted Urine Cytology for Noninvasive Detection of Muscle‐Invasive Urothelial Carcinoma: A Multi‐Center Diagnostic Study with Prospective Validation
Source: Adv Sci (Weinh). 2025 Jul 25;12(39):e08977. doi: 10.1002/advs.202508977 (PMC12533399; doi:10.1002/advs.202508977)
Supplement: Supplementary file 1 — Supporting Information [file ADVS-12-e08977-s001.docx]

**Supplementary Materials**

[**Supplementary Methods** 2](#_Toc202462943)

[**The process of prospective trial** 2](#_Toc202462944)

[**Preparatory technique method for making the slides** 2](#_Toc202462945)

[**Details of whole slide images scanning** 3](#_Toc202462946)

[**Strategies for dealing with imbalanced data** 3](#_Toc202462947)

[**Details of the TNF model** 4](#_Toc202462948)

[**Details of the network training process** 4](#_Toc202462949)

[**Supplementary Tables** 7](#_Toc202462950)

[**Supplementary Table 1. Performance of the PUCAS-M in MIUC detection in validation cohorts** 7](#_Toc202462951)

[**Supplementary Table 3. Sensitivity and AUROC of PUCAS-M in radiology Tx subgroup in validation cohorts** 9](#_Toc202462952)

[**Supplementary Table 4. Performance of the mPUCAS-M in MIUC detection in validation cohorts** 10](#_Toc202462953)

[**Supplementary Table 5. Comparison of sensitivity and AUROC of mPUCAS-M, PUCAS-M and radiologist assessments (MR/CT) in validation cohorts** 11](#_Toc202462954)

[**Supplementary Table 6. NRI and IDI of mPUCAS-M compared with PUCAS-M and radiologist assessments (MR/CT) in validation cohorts** 12](#_Toc202462955)

[**Supplementary Table 7. Sensitivity and AUROC of mPUCAS-M in radiology Tx subgroup in validation cohorts** 13](#_Toc202462956)

[**Supplementary Table 8. Comparison of sensitivity and AUROC of mPUCAS-M, PUCAS-M and radiologist assessments (MR/CT) in detecting MIBC and MI UTUC in different clinical scenarios** 14](#_Toc202462957)

[**Supplementary Figures** 15](#_Toc202462958)

[**Supplementary Figure 1. Representative examples for each diagnostic category that were used for annotation** 15](#_Toc202462959)

[**Supplementary Figure 2. The algorithm diagram of PUCAS-M** 16](#_Toc202462960)

[**Supplementary Figure 3. The algorithm diagram of the patch feature extraction stage** 18](#_Toc202462961)

[**Supplementary Figure 4. The algorithm diagram of the WSI- level diagnosis stage** 19](#_Toc202462962)

[**Supplementary Figure 5. ROC curves and sensitivity of the PUCAS-M in different cytology results** 20](#_Toc202462963)

[**Supplementary Figure 6. Examples of heatmaps of urine cytology WSIs in different MI status and cytology subgroups** 21](#_Toc202462964)

[**Supplementary Figure 7. Examples of MIUC diagnoses by PUCAS-M in non-MI and MI, with corresponding radiology and pathology images** 22](#_Toc202462965)

[**Supplementary Figure 8. DCA curves of the mPUCAS-M in validation cohorts** 23](#_Toc202462966)

[**Supplementary Figure 9. SHAP value of the mPUCAS-M** 24](#_Toc202462967)

[**Supplementary Figure 10. mPUCAS-M to detect MIBC after neoadjuvant treatment** 25](#_Toc202462968)

[**Supplementary Figure 11. One example of mPUCAS-M to detect hard-to-detect MI UTUC in initial diagnosis** 27](#_Toc202462969)

# **Supplementary Methods**

## **The process of prospective trial**

The slides collection in prospective trial in this study was conducted from July 7, 2023 to September 15, 2023, which included Sun Yat-sen Memorial Hospital of Sun Yat-Sen University (SYSMH) and Shen-Shan Central Hospital (SSCH). The inclusion criteria included patients (age > 18) who underwent liquid-based voided urine cytology examinations for initial diagnosis or recurrence detection, with or without urinary tract symptoms. The exclusion criteria included those who declined urothelial carcinoma (UC)-associated surgery and without histopathology reports despite radiology or urine cytology indicating the need for it; lack of radiology reports (MR or CT); and those with rare subtypes of tumours of the urinary tract, such as squamous cell carcinoma or adenocarcinoma. And the low-quality slides owing to low cell numbers (less than 20), extreme fading, and low-resolution whole-slide images (WSIs) caused by poor scanning quality were also excluded.

To mitigate the selection bias, the collection process of the golden standard and the artificial intelligence (AI) prediction was designed to be independent and blinded to each other. The outcome (T stage) was withheld from AI operators until after the prediction was made. To save time, slides that passed the quality control process were simultaneously sent for AI diagnosis. The final AI diagnosis results were saved on an encrypted hard disk, which was managed by the data regulatory committee for secure storage. These results were kept blinded from cytopathologists and clinicians to avoid any impact on normal clinical workflows.

The WSI-level golden standard labels were derived from histopathological reports. Gold standard of muscle invasive (MI) was based on the histopathological reports of transurethral resection of bladder tumour (TURBT), radical cystectomy, or radical nephroureterectomy. And the final follow-up was ended on December 15, 2023.

Until December 17, 2023, all the AI results and follow-up results (120 slides) were collected completely, and the prospective trail was unblinded. Subsequent statistical analyses were conducted to investigate the diagnostic performance of the Precision Urine Cytology AI Solution for Muscle-invasion (PUCAS-M).

## **Preparatory technique method for making the slides**

Urine cytology is primarily prepared using the sedimentation-based liquid-based slide method combined with Papanicolaou staining. For each urine sample with a unique ID identifier, the process begins with a 30-second shake on vortex generator (Vortex-Genie2, Scientific Industries, INC.) to ensure homogeneity. Subsequently, 8 ml of the supernatant is aspirated and transferred into a centrifuge tube containing 4 ml of separation extraction solution. The sample is then subjected to two cycles of centrifugation on low-speed centrifuge (JW-1048, Anhui Jiawen Equipment Industry Co., Ltd.). In the first centrifugation step, the speed is set at 600G for a duration of 5 minutes, following which 8 ml of the supernatant is carefully removed. In the second centrifugation step, the speed is again set at 600G, but for an extended duration of 10 minutes. Post-centrifugation, the supernatant is discarded, and the remaining pellet is resuspended by a 30-second shake on vortex generator.

Cell suspension is prepared using a PrepStain liquid-based slide preparation system (TriPath Imaging, INC.), and the automated staining is performed with hematoxylin and EA/OA stainers. An appropriate volume of the processed sample is aspirated into the PrepStain system, where cells are automatically deposited onto glass slides to form a diagnostic area of 13 mm in diameter. After cell deposition and staining, the slides are dehydrated for approximately 10 seconds in anhydrous alcohol and then for 5 minutes in a clearing agent, followed by immersion in a neutral resin for sealing.

The prepared slides are then placed into the digital slide scanner to capture the entire slide image, facilitating conversion to a digital format. This digital image is subsequently analysed using PUCAS-M.

## **Details of whole slide images scanning**

For model training and validation, all patient slides stained with the Papanicolaou method are scanned as whole-slide images (WSIs) at 40× magnification. To ensure the robustness of PUCAS-M, five distinct scanner models with varying technical specifications are utilized:

1. The PRECICE 600 digital slide scanner (UNIC TECHNOLOGIES, INC.), featuring a specimen-level pixel resolution of 0.25 μm × 0.25 μm;

2. The KF-PRO-400-HI (Ningbo Jiangfeng Bio-Information Technology Co., Ltd.) and the SQS-600P (Shenzhen Shengqiang Technology Co., Ltd.), both providing a pixel resolution of 0.24 μm × 0.24 μm;

3. The Pannoramic SCAN II (3DHISTECH Co., Ltd.), with a resolution of 0.27 μm × 0.27 μm;

4. The Win Medical Win60 (Zhiying Medical Technology, Jinan, China), offering a resolution of 0.25 μm × 0.25 μm.

This multi-scanner approach was implemented to minimize potential bias associated with single-device imaging variations.

## **Strategies for dealing with imbalanced data**

In preparation for the training process of patch feature extraction models, pathologists are tasked with sampling and annotating hundreds of patches from positive WSIs (≥ pT2). Meanwhile, all patches from negative WSIs are partitioned as either background or negative samples. However, it is important to note that the number of negative samples far outweighs that of positive ones, giving rise to potential bias in the analysis of these data. To mitigate the bias arising from an imbalanced dataset, equal weighted sampling techniques are employed.

## **Details of the TNF model**

In classical multi-instance Learning (MIL) settings, the samples are considered as bags, and models are designed to classify a bag of instances as positive or negative. In this approach, all instances share the same sample feature encoder. Subsequently, aggregation operators, typically "mean" or "max", are applied to the encoded features to generate a single bag representation for each sample. Finally, a dense layer is used to determine the probability of the bag being positive or negative.

To address the requirements of a computer-aided diagnosis system, we employ a two-layer bagging structure, where each sample is considered as a bag of sub-bags. The first layer consists of "patch-class" sub-bags. For each class, except the background class, we select N patches with the highest confidence and assign them to the corresponding "patch-class" sub-bag. As a result, we have a total of six sub-bags, with a maximum of N instances in each, for every WSI. These six sub-bags constitute the second layer of bags.

In TNF model, instances are passed through a feature encoder consisting of two dense layers. Instances within the same sub-bags share the same weight in the feature encoder. Subsequently, a gated version of attention-based pooling, originally introduced by Ilse et al. ^1^, is applied to each sub-bag in order to obtain their representations. The attention weights are calculated from the feature embeddings using a network defined by equation [1]:


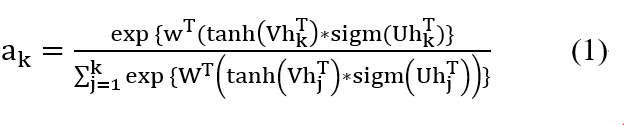


The sub-bag feature representation is obtained by computing the weighted average of the feature embedding, as defined in equation [2]:


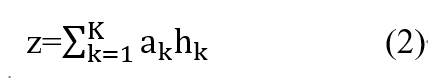


After the six sub-bags' feature representations is obtained, they are fed into another feature extractor that shares the same weights. Another attention weight is then calculated based on the encoded sub-bag features. Instead of computing the weighted average of the sub-bag features, the attention weight, followed by a concatenate operator to obtain the final bag representation, is applied. Finally, we add a dense layer to obtain the final class predicting logits from the final bag representation.

TNF leverages the attention mechanism and the permutation-invariant nature of patch instances. A Sub-bag for each patch-level class is created and used to form the final bag. The attention weights are solely calculated based on the feature itself, ensuring equal treatment of each patch-level class regardless of their number. Moreover, we apply the attention mechanism multiple times to emphasize the most potentially malignant instances.

## **Details of the network training process**

All the models in PUCAS-M were trained and validated using Python 3.8 and PyTorch 1.12.1 implemented on the CentOS Linux release 7.9.2009 operating system.

During the patch feature extraction model training stage, YOLOv7 ^2^, an advanced object detection model renowned for its superior trade-off between speed and accuracy, was meticulously trained to identify atypical cells within these patches. While ConvNeXt-B ^3^, an innovative convolution-based classification network at the forefront of research, was trained to discern the presence of atypical cells across the entirety of a given patch. The final network, RegNetY ^4^, an innovative architecture designed by researchers based on function space theory, was likewise trained to determine the presence of atypical cells across the entirety of each patch. All of these were initially pretrained on the ImageNet dataset ^5^, utilizing four NVIDIA GeForce RTX 3090 GPUs and two types of optimizers. YOLOv7 employed the Stochastic Gradient Descent (SGD) optimizer, starting with an initial learning rate of 0.01, while RegNetY and ConvNeXt used the Rectified Adam (RAdam) optimizer with a learning rate of 0.001. The batch sizes were set to 32 for YOLOv7, 48 for RegNetY and 64 for ConvNeXt. The optimal models were determined based on the parameter optimization set of patches.

In the training phase of patch feature extraction models, various data augmentation methods are employed to address the issues of model over-fitting and enhance its robust against staining variations and different scanners. These methods include Gaussian noise ^6^, ISO noise, multiplicative noise, image compression, RGB random shift, random brightness contrast, pixel dropout, blur, rotation, and flipping. Additionally, to enable YOLOv7 to identify objects at a smaller scale, Mosaic is incorporated into the training process, thereby reducing the dependence on a large mini-batch size. The patches used for YOLOv7 are standardized to a resolution of 640×640 pixels and are transformed into bounding boxes for atypical cells and features derived from the backbone network. Similarly, for RegNetY and ConvNeXt, the patches are resized to 1024×1024 and serve as a classifier of whether they contain atypical cells. The obtained bounding boxes and confidence from each network are utilized to calculate the loss, which is used to update the model weights along with the annotations. The algorithm diagram of the patch feature extraction process is shown in **Supplementary Fig.3**.

During WSI-diagnosis stage, three diagnosis models, namely the Transformer, attention-based Bi-LSTM ^7^, and TNF, were used. The attention-based Bi-LSTM model, which was a highly refined version of Bi-LSTM, treated all the patch results as a long-term fixed-size feature sequence and effectively extracted maximum relevant information from the sequence while eliminating noise. The Transformer model, which integrated multiple layers of self-attention mechanism and feed-forward neural networks, offered a comprehensive global modelling of feature sequences that captured the complex relationships between inputs. The TNF model, which employed a two-layer bagging structure based on multiple-instance learning and an attention mechanism, focused on the most promising malignancies. The three models were trained on 2 NIVIDA GeForce RTX 3090 GPUs with batch size of 128, 128 and 512 respectively. The Transformer and the attention-based Bi-LSTM utilized the advanced RAdam optimizer, with the initial learning rate of 0.0002 and 0.001 respectively, while TNF leveraged Adam with the learning rate of 0.0001 ^8^. The model weights of both Transformer and TNF are updated by the multi-class Cross Entropy Loss, which is expressed as equation [3]:


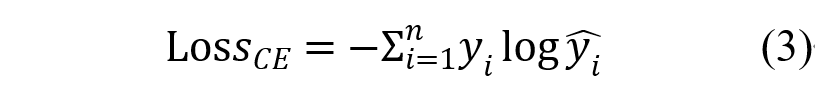


where $n$ denotes the total number of classes, $y_{i}$ represents the ground truth label for the current class, $\hat{y_{i}}$ denotes the probability of the current class.

The attention-based Bi-LSTM leverages Huber Loss function instead of Cross Entropy Loss. As is recognized, the process of HGUC is characterized by its continuous nature. Accordingly, a regression method was deemed necessary to accurately model the differing distances between distinct classes. Specifically, it is essential that the distance between the HGUC group and the NHGUC group surpasses the distance between the HGUC group and the AUC group, as well as the distance between the NHGUC group and the AUC group. To achieve this objective, the adoption of Huber Loss was deemed necessary to effectively capture the distances among the classes. The Huber Loss function (equation [4]), represented as follows, was selected to represent the inter-class distances:


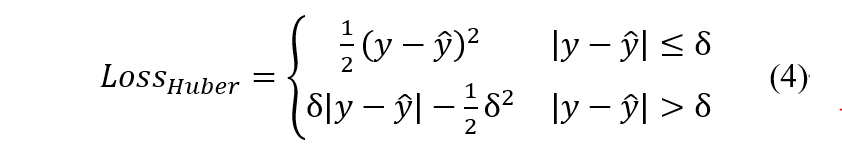


in which $y$ denotes the ground truth value of a given sample, while $\hat{y}$ represents the corresponding prediction generated by the model. Additionally, $\delta$ is a constant in determining the two distinct calculation approaches. When the bias between the prediction and the ground truth surpasses$\delta$, the loss assumes a linear nature. This characteristic is vital in mitigating the influence of outliers and enhancing the robustness of models in the training process. Conversely, when the discrepancy is smaller than delta, the loss is computed using the mean squared error, further bolstering the model's resistance to outliers.

As the above equation shown, the longer the distance between the prediction and the ground truth, the greater the loss. Consequently, the implementation of the loss function enabled the attention-based Bi-LSTM model to effectively extract the most salient and discriminating features from easily identifiable samples, such as NHGUC and HGUC, at the begin of training, thereby expediting model convergence. Moreover, noise samples – specifically those that were subjected to subjective labelling biases (primarily AUC) – incurred relatively low levels of loss due to their central positioning. As the result of these advantages, the influence caused by noise samples was considerably mitigated during the feature-learning process, thus enhancing the overall performance of the model. The algorithm diagram of the WSI-diagnosis process is shown in **Supplementary Fig.4**.

# **Supplementary Tables**

## **Supplementary Table 1. Performance of the PUCAS-M in MIUC detection in validation cohorts**

|  | Sensitivity (95% CI) | Specificity (95% CI) | Accuracy (95% CI) | Positive predictive value (95% CI) | Negative predictive value (95% CI) |
| --- | --- | --- | --- | --- | --- |
| **Validation Cohort (N = 733)** | 0.855 (0.791, 0.906) | 0.857 (0.826, 0.885) | 0.857 (0.829, 0.881) | 0.624 (0.556, 0.688) | 0.955 (0.934, 0.971) |
| Retrospective Validation Cohorts **(N = 613)** | 0.844 (0.769, 0.902) | 0.849 (0.815, 0.880) | 0.848 (0.817, 0.876) | 0.597 (0.521, 0.669) | 0.954 (0.929, 0.971) |
| Prospective Validation Cohorts **(N = 120)** | 0.903 (0.742, 0.980) | 0.899 (0.817, 0.953) | 0.900 (0.832, 0.947) | 0.757 (0.588, 0.882) | 0.964 (0.898, 0.992) |
| **Urothelial Carcinoma Classification** |  |  |  |  |  |
| Bladder Cancer **(N = 515)** | 0.818 (0.704, 0.902) | 0.869 (0.834, 0.898) | 0.862 (0.829, 0.891) | 0.478 (0.383, 0.574) | 0.970 (0.948, 0.984) |
| UTUC **(N = 218)** | 0.882 (0.798, 0.939) | 0.816 (0.737, 0.880) | 0.844 (0.789, 0.890) | 0.781 (0.690, 0.856) | 0.903 (0.832, 0.950) |
| **Age** |  |  |  |  |  |
| ≥ 65 **(N = 415)** | 0.865 (0.776, 0.928) | 0.850 (0.806, 0.887) | 0.853 (0.815, 0.886) | 0.611 (0.520, 0.697) | 0.958 (0.929, 0.978) |
| < 65 **(N = 318)** | 0.843 (0.736, 0.919) | 0.867 (0.818, 0.907) | 0.862 (0.819, 0.898) | 0.641 (0.535, 0.739) | 0.951 (0.915, 0.975) |
| **Gender** |  |  |  |  |  |
| Male **(N = 574)** | 0.836 (0.758, 0.897) | 0.874 (0.840, 0.903) | 0.866 (0.835, 0.893) | 0.642 (0.562, 0.716) | 0.952 (0.927, 0.970) |
| Female **(N = 159)** | 0.919 (0.781, 0.983) | 0.795 (0.713, 0.863) | 0.824 (0.756, 0.880) | 0.576 (0.441, 0.704) | 0.970 (0.915, 0.994) |
| **Clinical Scenario Subgroups** |  |  |  |  |  |
| Initial diagnosis **(N = 565)** | 0.872 (0.797, 0.926) | 0.848 (0.812, 0.880) | 0.853 (0.821, 0.881) | 0.600 (0.522, 0.674) | 0.962 (0.938, 0.979) |
| Recurrence Detection **(N = 168)** | 0.810 (0.659, 0.914) | 0.889 (0.821, 0.938) | 0.869 (0.808, 0.916) | 0.708 (0.559, 0.830) | 0.933 (0.873, 0.971) |
| Neoadjuvant Treatment **(N = 67)** | 0.828 (0.642, 0.942) | 0.868 (0.719, 0.956) | 0.851 (0.743, 0.926) | 0.828 (0.642, 0.942) | 0.868 (0.719, 0.956) |

The above analyses were conducted in validation cohorts and their subgroups.

PUCAS-M, The Precision Urine Cytology AI Solution for Muscle-invasion. MIUC, Muscle Invasive Urothelial Carcinoma. CI, Confidence Interval. UTUC, Upper-Tract Urothelial Carcinoma.

**Supplementary Table 2. Comparison of sensitivity and AUROC of PUCAS-M and radiologist assessments (including CT and MR separately) in validation cohorts**

|  | Sensitivity of PUCAS-M (95% CI) | AUROC of PUCAS-M  (95% CI) | Sensitivity of Radiologists (95% CI) | AUROC of Radiologists  (95% CI) | Sensitivity of MR (95% CI) | AUROC of MR (95% CI) | Sensitivity of CT (95% CI) | AUROC of CT  (95% CI) |
| --- | --- | --- | --- | --- | --- | --- | --- | --- |
| **Validation Cohort (N = 733)** | 0.855 (0.791, 0.906) | 0.857 (0.820-0.895) | 0.699 (0.606, 0.782) ** | 0.773 (0.727-0.818) * | 0.731 (0.590, 0.844) * | 0.783 (0.717-0.848) * | 0.672 (0.540, 0.787) ** | 0.764 (0.701-0.827) |
| **Retrospective Validation Cohorts (N = 613)** | 0.844 (0.769, 0.902) | 0.853 (0811-0.895) | 0.709 (0.601, 0.802) * | 0.780 (0.729-0.831) | 0.730 (0.559, 0.862) | 0.780 (0.702-0.857) * | 0.694 (0.546, 0.817) * | 0.780 (0.711-0.849) |
| **Prospective Validation Cohorts (N = 120)** | 0.903 (0.742, 0.980) | 0.882 (0.799-0.965) | 0.667 (0.460, 0.835) * | 0.744 (0.644-0.845) * | 0.733 (0.449, 0.922) | 0.797 (0.668-0.926) | 0.583 (0.277, 0.848) * | 0.684 (0.523-0.844) ** |
| **Urothelial Carcinoma Classification** |  |  |  |  |  |  |  |  |
| Bladder Cancer **(N = 515)** | 0.818 (0.704, 0.902) | 0.829 (0.766-0.892) | 0.639 (0.506, 0.758) * | 0.748 (0.684-0.811) * | 0.743 (0.567, 0.875) | 0.789 (0.712-0.867) | 0.500 (0.299, 0.701) ** | 0.688 (0.588-0.789) * |
| UTUC **(N = 218)** | 0.882 (0.798, 0.939) | 0.864 (0.809-0.918) | 0.769 (0.632, 0.875) | 0.788 (0.718-0.858) | 0.706 (0.440, 0.897) | 0.765 (0.619-0.910) * | 0.800 (0.631, 0.916) | 0.801 (0.721-0.881) |
| **Clinical Scenario Subgroups** |  |  |  |  |  |  |  |  |
| Initial diagnosis **(N = 565)** | 0.872 (0.797, 0.926) | 0.871 (0.832, 0.911) | 0.740 (0.624, 0.835) * | 0.800 (0.747-0.854) * | 0.741 (0.537, 0.889) | 0.790 (0.701-0.879) * | 0.739 (0.589, 0.857) * | 0.808 (0.740-0.875) |
| Recurrence Detection **(N = 168)** | 0.810 (0.659, 0.914) | 0.820 (0.730-0.911) | 0.625 (0.458, 0.773) | 0.711 (0.626-0.795) * | 0.720 (0.506, 0.879) | 0.771 (0.668-0.874) | 0.467 (0.213, 0.734) * | 0.619 (0.478-0.761) |
| Neoadjuvant Treatment **(N = 67)** | 0.828 (0.642, 0.942) | 0.789 (0.661-0.916) | 0.643 (0.441, 0.814) | 0.607 (0.484-0.730) ** | 0.765 (0.501, 0.932) | 0.698 (0.546-0.851) | 0.455 (0.167, 0.766) * | 0.523 (0.323-0.722) |

The above analyses were conducted in validation cohorts and their subgroups. The comparison of sensitivity was assessed by Pearson’s χ^2^ test. The comparison of AUROC was assessed by Delong test. *P < 0·05, **P < 0·01, ***P < 0·001.

PUCAS-M, The Precision Urine Cytology AI Solution for Muscle-invasion. CT, computed tomography. MR, magnetic resonance. UTUC, Upper-Tract Urothelial Carcinoma. AUROC, Area Under the Receiver Operating Characteristic. CI, Confidence Interval.

## **Supplementary Table 3. Sensitivity and AUROC of PUCAS-M in radiology Tx subgroup in validation cohorts**

|  | Sensitivity of PUCAS-M (95% CI) | AUROC of PUCAS-M (95% CI) |
| --- | --- | --- |
| **Validation Cohort (N = 113)** | 0.935 (0.821, 0.986) | 0.814 (0.727-0.901) |
| Retrospective Validation Cohorts **(N = 93)** | 0.929 (0.805, 0.985) | 0.782 (0.679-0.885) |
| Prospective Validation Cohorts **(N = 20)** | 1.000 (0.398, 1.000) | 0.938 (0.815-1.000) |
| **Urothelial Carcinoma Classification** |  |  |
| Bladder Cancer **(N = 45)** | 0.800 (0.284, 0.995) | 0.750 (0.434-1.000) |
| UTUC **(N = 68)** | 0.951 (0.835, 0.994) | 0.739 (0.598-0.881) |
| **Clinical Scenario Subgroups** |  |  |
| Initial diagnosis **(N = 98)** | 0.932 (0.813, 0.986) | 0.798 (0.702-0.895) |
| Recurrence Detection **(N = 15)** | 1.000 (0.158, 1.000) | 0.923 (0.772-1.000) |
| Neoadjuvant Treatment **(N = 4)** | 1.000 (0.025, 1.000) | 1.000 (NA, NA) |

The above analyses were conducted in validation cohorts and their subgroups. The comparison of sensitivity was assessed by Pearson’s χ^2^ test. The comparison of AUROC was assessed by Delong test. *P < 0·05, **P < 0·01, ***P < 0·001.

PUCAS-M, The Precision Urine Cytology AI Solution for Muscle-invasion. UTUC, Upper-Tract Urothelial Carcinoma. AUROC, Area Under the Receiver Operating Characteristic. CI, Confidence Interval.

## **Supplementary Table 4. Performance of the mPUCAS-M in MIUC detection in validation cohorts**

|  | Sensitivity (95% CI) | Specificity (95% CI) | Accuracy (95% CI) | Positive predictive value (95% CI) | Negative predictive value (95% CI) |
| --- | --- | --- | --- | --- | --- |
| **Validation Cohort (N = 733)** | 0.874 (0.812, 0.921) | 0.864 (0.833, 0.891) | 0.866 (0.840, 0.890) | 0.641 (0.573, 0.704) | 0.961 (0.941, 0.976) |
| Retrospective Validation Cohorts **(N = 613)** | 0.867 (0.796, 0.921) | 0.862 (0.828, 0.891) | 0.863 (0.833, 0.889) | 0.624 (0.548, 0.695) | 0.961 (0.938, 0.977) |
| Prospective Validation Cohorts **(N = 120)** | 0.903 (0.742, 0.980) | 0.876 (0.790, 0.937) | 0.883 (0.812, 0.935) | 0.718 (0.551, 0.850) | 0.963 (0.896, 0.992) |
| **Urothelial Carcinoma Classification** |  |  |  |  |  |
| Bladder Cancer **(N = 515)** | 0.833 (0.721, 0.914) | 0.875 (0.841, 0.904) | 0.870 (0.838, 0.898) | 0.495 (0.399, 0.592) | 0.973 (0.952, 0.986) |
| UTUC **(N = 218)** | 0.903 (0.824, 0.955) | 0.824 (0.746, 0.886) | 0.858 (0.804, 0.901) | 0.792 (0.703, 0.865) | 0.920 (0.853, 0.963) |
| **Age** |  |  |  |  |  |
| ≥ 65 **(N = 415)** | 0.876 (0.790, 0.937) | 0.847 (0.803, 0.884) | 0.853 (0.815, 0.886) | 0.609 (0.519, 0.694) | 0.962 (0.932, 0.981) |
| < 65 **(N = 318)** | 0.871 (0.770, 0.939) | 0.887 (0.841, 0.924) | 0.884 (0.843, 0.917) | 0.685 (0.578, 0.780) | 0.961 (0.927, 0.982) |
| **Gender** |  |  |  |  |  |
| Male **(N = 574)** | 0.852 (0.777, 0.910) | 0.874 (0.840, 0.903) | 0.869 (0.839, 0.896) | 0.646 (0.567, 0.720) | 0.956 (0.932, 0.974) |
| Female **(N = 159)** | 0.946 (0.818, 0.993) | 0.828 (0.749, 0.890) | 0.855 (0.791, 0.906) | 0.625 (0.485, 0.751) | 0.981 (0.932, 0.998) |
| **Clinical Scenario Subgroups** |  |  |  |  |  |
| Initial diagnosis **(N = 565)** | 0.889 (0.817, 0.939) | 0.862 (0.826, 0.892) | 0.867 (0.836, 0.894) | 0.627 (0.548, 0.700) | 0.967 (0.945, 0.983) |
| Recurrence Detection **(N = 168)** | 0.833 (0.686, 0.930) | 0.873 (0.802, 0.926) | 0.863 (0.802, 0.911) | 0.686 (0.541, 0.809) | 0.940 (0.881, 0.976) |
| Neoadjuvant Treatment **(N = 67)** | 0.828 (0.642, 0.942) | 0.816 (0.657, 0.923) | 0.821 (0.708, 0.904) | 0.774 (0.589, 0.904) | 0.861 (0.705, 0.953) |

The above analyses were conducted in validation cohorts and their subgroups.

mPUCAS-M, The Multi-modal Precision Urine Cytology AI Solution for Muscle-invasion. UTUC, Upper-Tract Urothelial Carcinoma. MIUC, Muscle Invasive Urothelial Carcinoma. CI, Confidence Interval.

## **Supplementary Table 5. Comparison of sensitivity and AUROC of mPUCAS-M, PUCAS-M and radiologist assessments (MR/CT) in validation cohorts**

|  | Sensitivity of mPUCAS-M (95% CI) | P Value compared to PUCAS-M | P Value Compared to Radiologists (MR/ CT) | AUROC of mPUCAS-M (95% CI) | P Value Compared to PUCAS-M | P Value Compared to Radiologists (MR/ CT) |
| --- | --- | --- | --- | --- | --- | --- |
| **Validation Cohort (N = 733)** | 0.874 (0.812, 0.921) | 0.623 | <0.001 ***  (MR: 0.015*/ CT: <0.001 ***) | 0.900 (0.870, 0.930) | 0.119 | <0.001 ***  (MR: 0.019 */  CT: 0.003 **) |
| Retrospective Validation Cohorts **(N = 613)** | 0.867 (0.796, 0.921) | 0.594 | 0.004 **  (MR: 0.047 */ CT:0.007 **) | 0.894 (0.860-0.928) | 0.065 | 0.007 **  (MR: 0.049 */  CT: 0.030 *) |
| Prospective Validation Cohorts **(N = 120)** | 0.903 (0.742, 0.980) | 1.000 | 0.027 *  (MR: 0.133/ CT:0.016 *) | 0.924 (0.863-0.986) | 0.415 | 0.018 *  (MR: 0.211/  CT: 0.022 *) |
| **Urothelial Carcinoma Classification** |  |  |  |  |  |  |
| Bladder Cancer **(N = 515)** | 0.833 (0.721, 0.914) | 0.819 | 0.013 *  (MR:0.278/  CT: 0.001 **) | 0.884 (0.834-0.934) | 0.305 | 0.008 **  (MR: 0.163/  CT: 0.004 **) |
| UTUC **(N = 218)** | 0.903 (0.824, 0.955) | 0.636 | 0.028 *  (MR:0.025 */  CT: 0.115) | 0.900 (0.858-0.942) | 0.258 | 0.075  (MR: 0.211/  CT: 0.185) |
| **Clinical Scenario Subgroups** |  |  |  |  |  |  |
| Initial diagnosis **(N = 565)** | 0.889 (0.817, 0.939) | 0.687 | 0.008 **  (MR: 0.045 */  CT: 0.017 *) | 0.906 (0.873-0.939) | 0.105 | 0.019 *  (MR: 0.079/  CT: 0.079) |
| Recurrence Detection **(N = 168)** | 0.833 (0.686, 0.930) | 0.776 | 0.033 *  (MR:0.270/  CT: 0.006 **) | 0.884 (0.819-0.948) | 0.819 | 0.009 **  (MR: 0.185/  CT: 0.004 **) |
| Neoadjuvant Treatment **(N = 67)** | 0.828 (0.642, 0.942) | 1.000 | 0.113  (MR:0.604/  CT:0.018 *) | 0.862 (0.770-0.953) | 0.416 | 0.007 **  (MR: 0.181/  CT: 0.011 *) |

The above analyses were conducted in validation cohorts and their subgroups. The comparison of sensitivity was assessed by Pearson’s χ^2^ test. The comparison of AUROC was assessed by Delong test. *P < 0·05, **P < 0·01, ***P < 0·001.

AUROC, Area Under the Receiver Operating Characteristic. PUCAS-M, The Precision Urine Cytology AI Solution for Muscle-invasion. mPUCAS-M, The Multi-modal Precision Urine Cytology AI Solution for Muscle-invasion. UTUC, Upper-Tract Urothelial Carcinoma. CI, Confidence Interval. CT, computed tomography. MR, magnetic resonance.

## **Supplementary Table 6. NRI and IDI of mPUCAS-M compared with PUCAS-M and radiologist assessments (MR/CT) in validation cohorts**

|  | NRI (95% CI) | | IDI (95% CI) | |
| --- | --- | --- | --- | --- |
|  | Compared to PUCAS-M | Compared to Radiologists (MR/CT) | Compared to PUCAS-M | Compared to Radiologists  (MR/CT) |
| **Validation Cohort (N = 733)** | 0.0258  [-0.0066 - 0.0583] | 0.18 [ 0.0673 - 0.2927] **  (MR: 0.203 [ 0.0444 - 0.3615] */  CT: 0.1617 [ 0.0025 - 0.3209] *) | 0.0258  [ -0.0067 - 0.0584] | 0.18 [ 0.0669 - 0.2932] **  (MR: 0.203 [ 0.043 - 0.3629] */  CT: 0.1617 [ 0.0013 - 0.3221] *) |
| Retrospective Validation Cohorts **(N = 613)** | 0.0358  [ -0.0021 - 0.0737] | 0.1509 [ 0.0192 - 0.2827] *  (MR: 0.2047 [ 0.0147 - 0.3948] */  CT: 0.1102 [ -0.0708 - 0.2912]) | 0.0358  [ -0.0022 - 0.0738] | 0.1509 [ 0.0185 - 0.2834] *  (MR: 0.2047 [ 0.0123 - 0.3971] */  CT: 0.1102 [ -0.0725 - 0.2929 ]) |
| Prospective Validation Cohorts **(N = 120)** | -0.0225  [ -0.0762 - 0.0313] | 0.2907 [ 0.0703 - 0.5111] **  (MR: 0.2167 [ -0.0727 - 0.5061]/  CT: 0.3874 [ 0.0641 - 0.7107] *) | -0.0225  [ -0.0765 - 0.0316] | 0.2907 [ 0.0668 - 0.5146] **  (MR: 0.2167 [ -0.0815 - 0.5148]/  CT: 0.3874 [ 0.0529 - 0.7219] *) |
| **Urothelial Carcinoma Classification** |  |  |  |  |
| Bladder Cancer **(N = 515)** | 0.0218  [ -0.0197 - 0.0634] | 0.2163 [ 0.0602 - 0.3724] **  (MR: 0.1389 [ -0.0562 -0.3339]/  CT: 0.3313 [ 0.0867 - 0.5759] **) | 0.0218  [ -0.0199 - 0.0636] | 0.2163 [ 0.059 - 0.3736] **  (MR: 0.1389 [ -0.0587 - 0.3364]/  CT: 0.3313 [ 0.0821 - 0.5805] **) |
| UTUC **(N = 218)** | 0.0295  [ -0.0214 - 0.0804] | 0.1676 [ -0.0114 - 0.3465]  (MR: 0.2941 [ -0.0618 - 0.65]/  CT: 0.2941 [ -0.0727 - 0.661 ]) | 0.0295  [ -0.0216 - 0.0806] | 0.1676 [-0.0128 - 0.348]  (MR: 0.0864 [ -0.1245 - 0.2973]/  CT: 0.0864 [ -0.127 - 0.2999 ]) |
| **Clinical Scenario Subgroups** |  |  |  |  |
| Initial diagnosis **(N = 565)** | 0.0305  [ -0.0063 - 0.0673] | 0.1385 [ 0.0102 - 0.2668] *  (MR: 0.2388 [ 0.045 - 0.4325] */  CT: 0.0737 [ -0.0941 - 0.2415]) | 0.0305  [ -0.0064 - 0.0674] | 0.1385 [ 0.0094 - 0.2676] *  (MR: 0.2388 [ 0.0418 - 0.4357] */  CT: 0.0737 [ -0.0958 - 0.2432 ]) |
| Recurrence Detection **(N = 168)** | 0.0079  [ -0.0594 - 0.0753] | 0.2796 [ 0.0594 - 0.4998] *  (MR: 0.1557 [ -0.1092 -0.4206]/  CT: 0.4561 [ 0.0763 - 0.836] *) | 0.0079  [ -0.06 - 0.0758] | 0.2796 [ 0.057 - 0.5023] *  (MR: 0.1557 [ -0.1139 - 0.4253]/  CT: 0.4561 [ 0.0643 - 0.848] *) |
| Neoadjuvant Treatment **(N = 67)** | -0.0526  [ -0.1779 - 0.0726] | 0.4357 [ 0.1209 - 0.7505] **  (MR: 0.2755 [ -0.1067 - 0.6577]/  CT: 0.6477 [ 0.1162 - 1.1793] *) | -0.0526  [ -0.1795 - 0.0743] | 0.4357 [ 0.1156 - 0.7558] **  (MR: 0.2755 [ -0.1178 - 0.6689]/  CT: 0.6477 [ 0.0928 - 1.2026] *) |

The above analyses were conducted in validation cohorts and their subgroups. *P < 0·05, **P < 0·01, ***P < 0·001.

NRI, Net Reclassification Improvement. IDI, Integrated Discrimination Improvement. UTUC, Upper-Tract Urothelial Carcinoma. CI, Confidence Interval. CT, computed tomography. MR, magnetic resonance. PUCAS-M, The Precision Urine Cytology AI Solution for Muscle-invasion. mPUCAS-M, The Multi-modal Precision Urine Cytology AI Solution for Muscle-invasion.

## **Supplementary Table 7. Sensitivity and AUROC of** **mPUCAS-M in radiology Tx subgroup in validation cohorts**

|  | Sensitivity of mPUCAS-M (95% CI) | AUROC of mPUCAS-M (95% CI) |
| --- | --- | --- |
| **Validation Cohort (N = 113)** | 0.935 (0.821, 0.986) | 0.854 (0.781-0.926) |
| Retrospective Validation Cohorts **(N = 93)** | 0.929 (0.805, 0.985) | 0.824 (0.735-0.912) |
| Prospective Validation Cohorts **(N = 20)** | 1.000 (0.398, 1.000) | 0.984 (0.941-1.000) |
| **Urothelial Carcinoma Classification** |  |  |
| Bladder Cancer **(N = 45)** | 0.800 (0.284, 0.995) | 0.835 (0.629-1.000) |
| UTUC **(N = 68)** | 0.951 (0.835, 0.994) | 0.769 (0.639-0.898) |
| **Clinical Scenario Subgroups** |  |  |
| Initial diagnosis **(N = 98)** | 0.932 (0.813, 0.986) | 0.843 (0.763-0.923) |
| Recurrence Detection **(N = 15)** | 1.000 (0.158, 1.000) | 0.923 (0.772-1.000) |
| Neoadjuvant Treatment **(N = 4)** | 1.000 (0.025, 1.000) | 1.000 (NA, NA) |

The above analyses were conducted in validation cohorts and their subgroups. The comparison of sensitivity was assessed by Pearson’s χ^2^ test. The comparison of AUROC was assessed by delong test. *P < 0·05, **P < 0·01, ***P < 0·001.

AUROC, Area Under the Receiver Operating Characteristic. PUCAS-M, The Precision Urine Cytology AI Solution for Muscle-invasion. mPUCAS-M, The Multi-modal Precision Urine Cytology AI Solution for Muscle-invasion. UTUC, Upper-Tract Urothelial Carcinoma. AUROC, Area Under the Receiver Operating Characteristic.

## **Supplementary Table 8. Comparison of sensitivity and AUROC of mPUCAS-M, PUCAS-M and radiologist assessments (MR/CT) in detecting MIBC and MI UTUC in different clinical scenarios**

|  | Sensitivity of mPUCAS-M (95% CI) | P Value compared to PUCAS-M | P Value Compared to Radiologists (MR/CT) | AUROC of mPUCAS-M (95% CI) | P Value Compared to PUCAS-M | P Value Compared to Radiologists (MR/CT) |
| --- | --- | --- | --- | --- | --- | --- |
| **Bladder Cancer** |  |  |  |  |  |  |
| Initial diagnosis **(N = 370)** | 0.828 (0.642, 0.942) | 1.000 | 0.140  (MR:0.568/CT:0.071) | 0.882 (0.806-0.959) | 0.005 ** | 0.050  (MR:0.586/CT:0.126) |
| Recurrence Detection **(N = 145)** | 0.838 (0.680, 0.938) | 0.760 | 0.044 *  (MR:0.512/CT:0.009**) | 0.883 (0.815-0.952) | 0.013 * | 0.007 **  (MR: 0.438/CT:0.173) |
| Neoadjuvant Treatment **(N = 55)** | 0.833 (0.626, 0.953) | 1.000 | 0.154  (MR: 0.792/CT:0.013*) | 0.865 (0.765-0.965) | 0.153 | 0.002**  (MR:0.193/CT:0.425) |
| **UTUC** |  |  |  |  |  |  |
| Initial diagnosis **(N = 195)** | 0.909 (0.829, 0.960) | 0.619 | 0.047 *  (MR: 0.110/CT: 0.293) | 0.898 (0.854-0.942) | 0.064 | 0.050  (MR: 0.044 */CT:0.687) |
| Recurrence Detection **(N = 23)** | 0.800 (0.284, 0.995) | 1.000 | 0.490  (MR: 0.427/CT:0.673) | 0.883 (0.667-1.000) | 0.089 | 0.146  (MR: 0.480/CT:0.976) |
| Neoadjuvant Treatment **(N = 12)** | 0.800 (0.284, 0.995) | 1.000 | 0.490  (MR: 0.427/CT:0.673) | 0.857 (0.604-1.000) | 0.075 | 0.385  (MR: 1.000/CT:0.818) |

The above analyses were conducted in validation cohorts and their subgroups. The comparison of sensitivity was assessed by Pearson’s χ^2^ test. The comparison of AUROC was assessed by delong test. *P < 0·05, **P < 0·01, ***P < 0·001.

AUROC, Area Under the Receiver Operating Characteristic. PUCAS-M, The Precision Urine Cytology AI Solution for Muscle-invasion. mPUCAS-M, The Multi-modal Precision Urine Cytology AI Solution for Muscle-invasion. CI, Confidence Interval. CT, computed tomography. MR, magnetic resonance. UTUC, Upper-Tract Urothelial Carcinoma. MI, Muscle Invasive. MIBC, Muscle Invasive Bladder Cancer.

# **Supplementary Figures**

## **Supplementary Figure 1. Representative examples for each diagnostic category that were used for annotation**


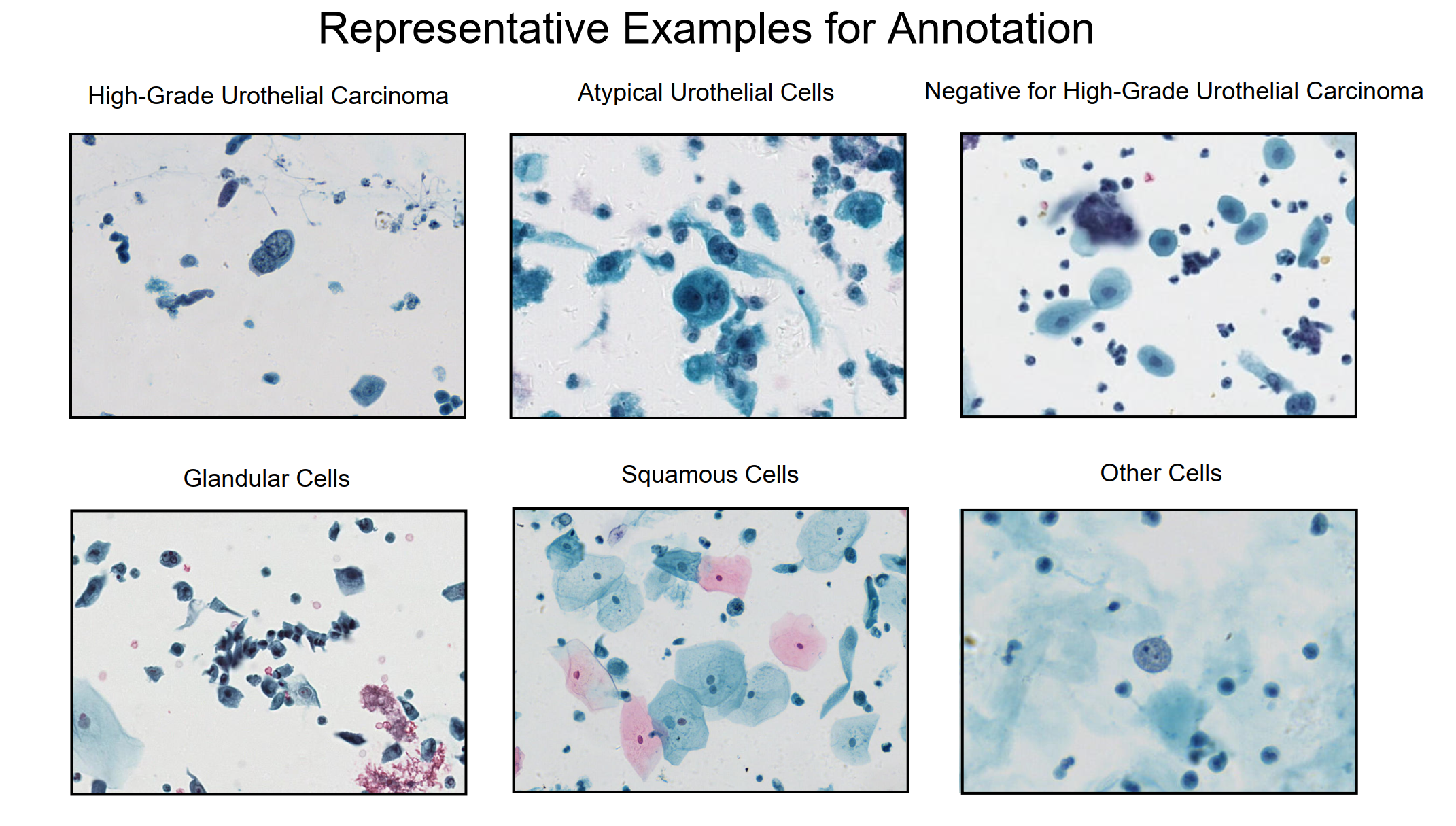


For model training, we obtained cell-level annotations from two expert cytopathologists, each with >15 years of experience in urine cytology diagnosis; they carefully reviewed and labelled urothelial cells into normal, atypical, or malignant categories following the established diagnostic criteria for TPS. Additionally, other cell types present in urine, such as glandular and squamous cells, were annotated. Uncertain cells identified by the cytopathologists and degenerated cells were annotated as ‘others’. Finally, 315,149 cells were annotated in the training cohort.

TPS, the Paris System.

## **Supplementary Figure 2. The algorithm diagram of PUCAS-M**

**
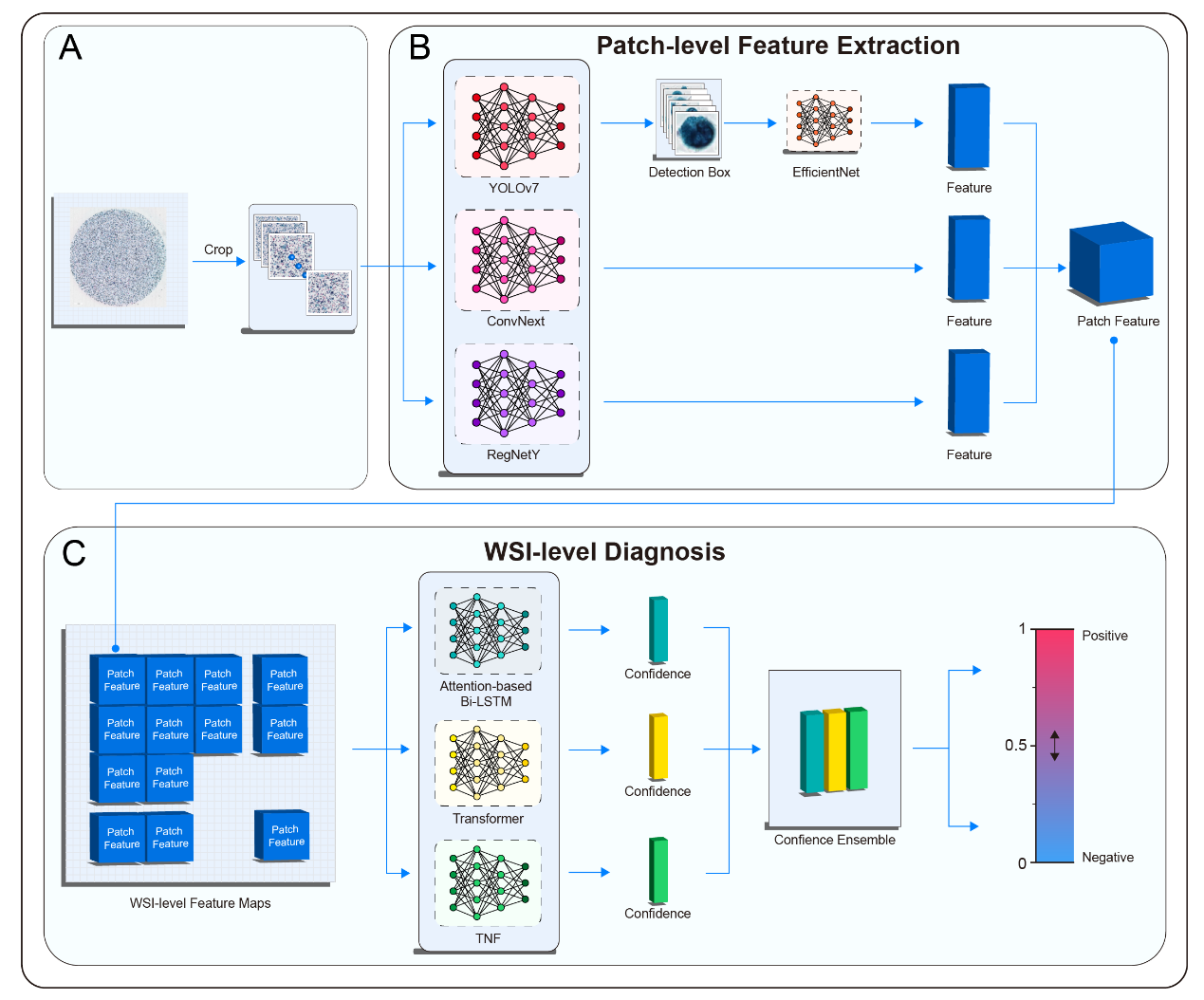
**

(A) The modelling process of PUCAS-M included three distinct stages. In the initial stage, the high-resolution WSIs, consisting of a remarkable 50,000*50,000 pixels, were divided into numerous non-overlapping patches using a sliding window approach. The crop size was carefully set to 1024*1024 pixels, corresponding to a spatial resolution of 0.25 micrometres per pixel (40 times magnification). (B) At the subsequent stage, individual patches extracted from the WSIs were subjected to patch-level feature extraction, which encompassed the integration of three distinct feature extraction models, including YOLOv7, EfficientNet, and ConvNext. YOLOv7 was meticulously trained to identify atypical cells within these patches. Concomitantly, these identified atypical cells were further classified by the highly efficacious EfficientNet. Additionally, RegNetY and ConvNeXt-B were trained to discern the presence of atypical cells across the entirety of a given patch. (C) The patch results generating from the same WSI were merged to form WSI-level feature maps that served as the input of the diagnosis stage. (D) The WSI-level diagnosis stage comprised three distinct models, namely attention-based Bi-LSTM, Transformer, and TNF model, that independently predicted the confidence of each class. (F) The final confidence of each class was determined by averaging the confidence scores derived from each WSI-level feature-extraction model, with appropriate weighting.

PUCAS-M, The Precision Urine Cytology AI Solution for Muscle-invasion. WSIs, whole slide images. Bi-LSTM, Bi-directional Long Short-Term Memory. TNF, Top-N Feature.

## **Supplementary Figure 3. The algorithm diagram of the patch feature extraction stage**

**
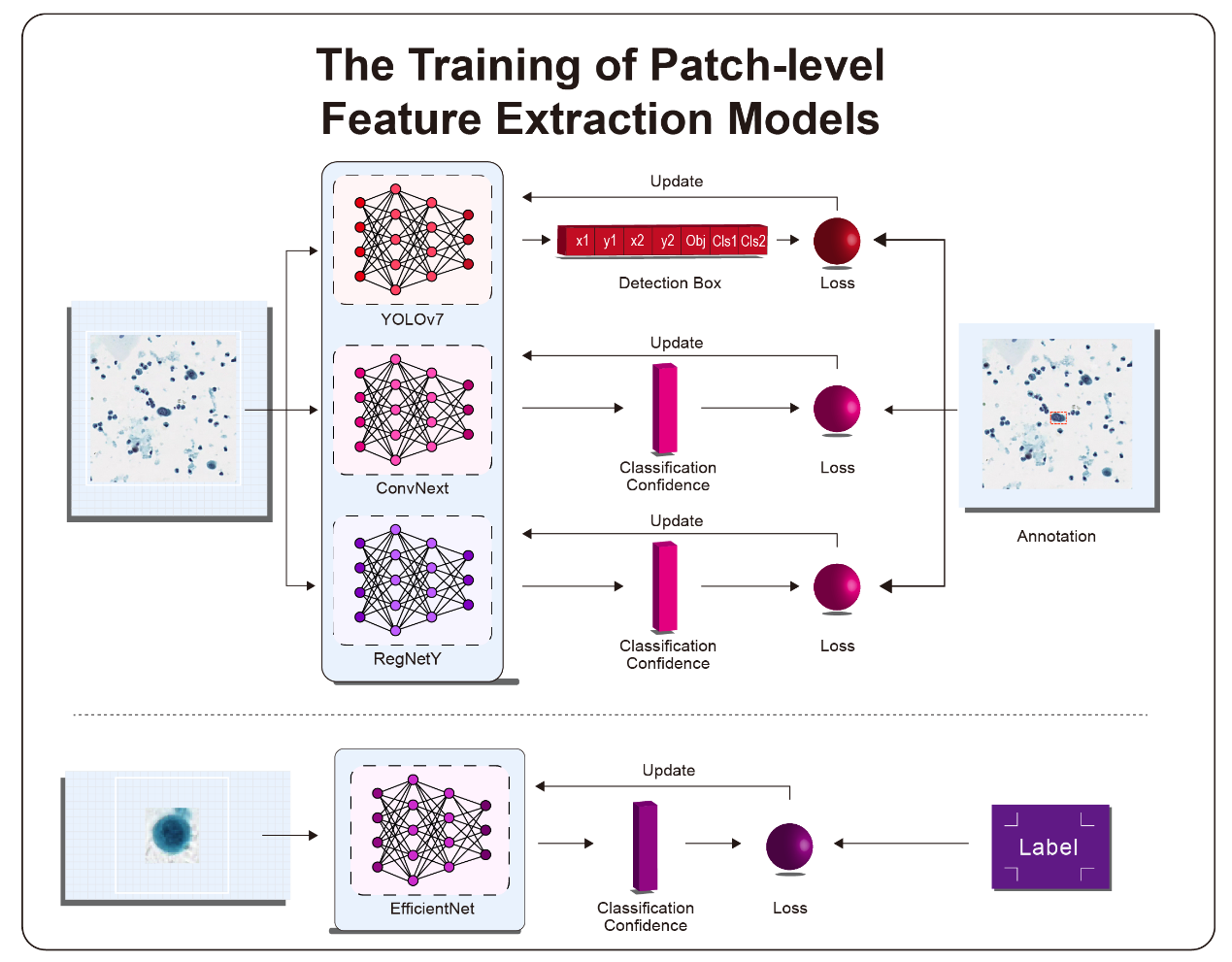
**

The patches used for YOLOv7 are standardized to a resolution of 640×640 pixels and are transformed into bounding boxes for atypical cells and features derived from the backbone network. Similarly, for RegNetY and ConvNeXt, the patches are resized to 1024×1024 and serve as a classifier of whether they contain atypical cells. The obtained bounding boxes and confidence from each network are utilized to calculate the loss, which is used to update the model weights along with the annotations.

## **Supplementary Figure 4. The algorithm diagram of the WSI- level diagnosis stage**

**
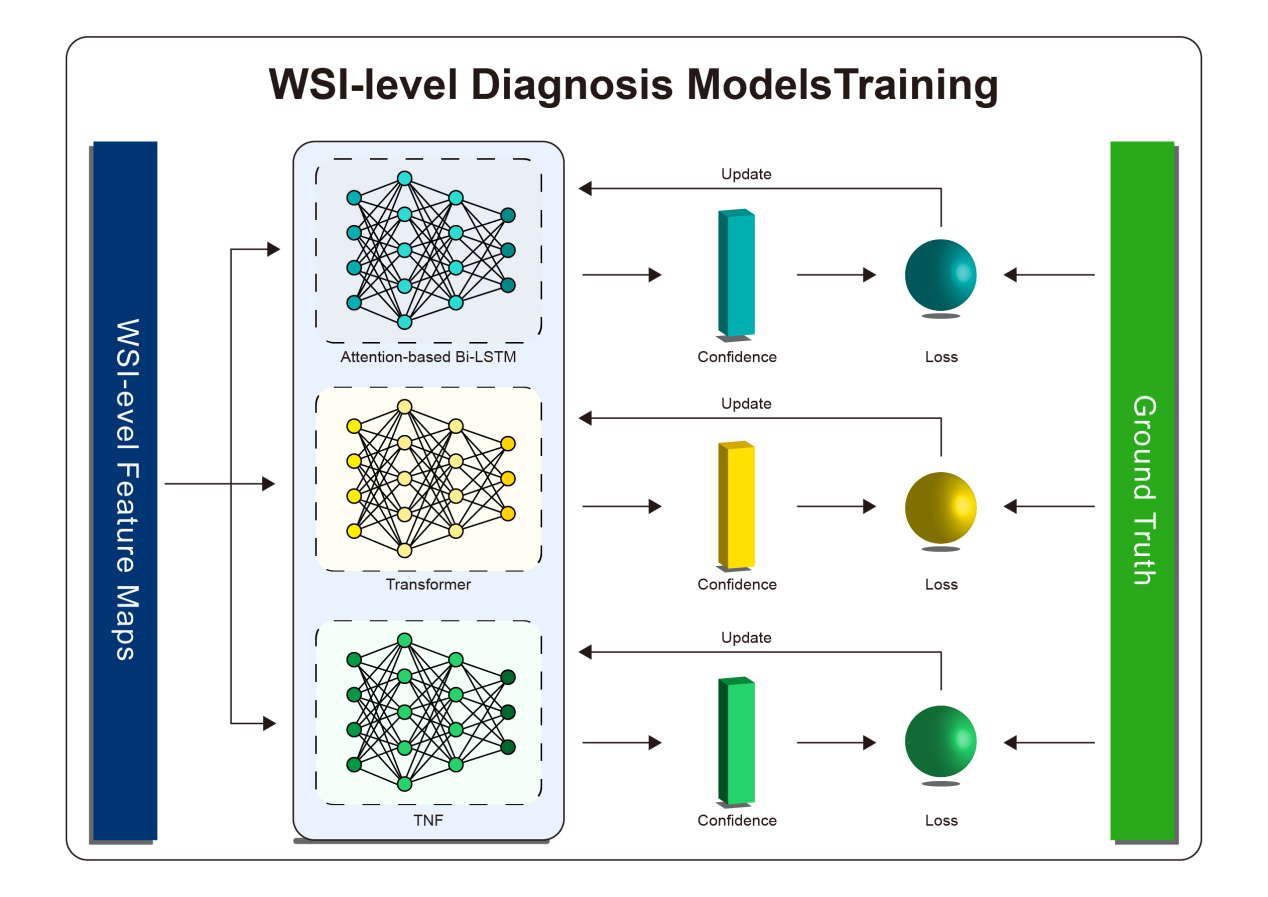
**

The three models were trained with batch size of 128, 128 and 512, respectively. The Transformer and the attention-based Bi-LSTM utilized the advanced RAdam optimizer, with the initial learning rate of 0.0002 and 0.001 respectively, while TNF leveraged Adam with a learning rate of 0.0001. The model weights of both Transformer and TNF were updated by the multi-class Cross Entropy Loss. The model weights of Bi-LSTM were updated by the Huber Loss function, enabling the attention-based Bi-LSTM model to effectively extract the most salient and discriminating features from easily identifiable samples, thereby expediting model convergence.

WSIs, Whole Slide Images. Bi-LSTM, Bi-directional Long Short-Term Memory. TNF, Top-N Feature.

##
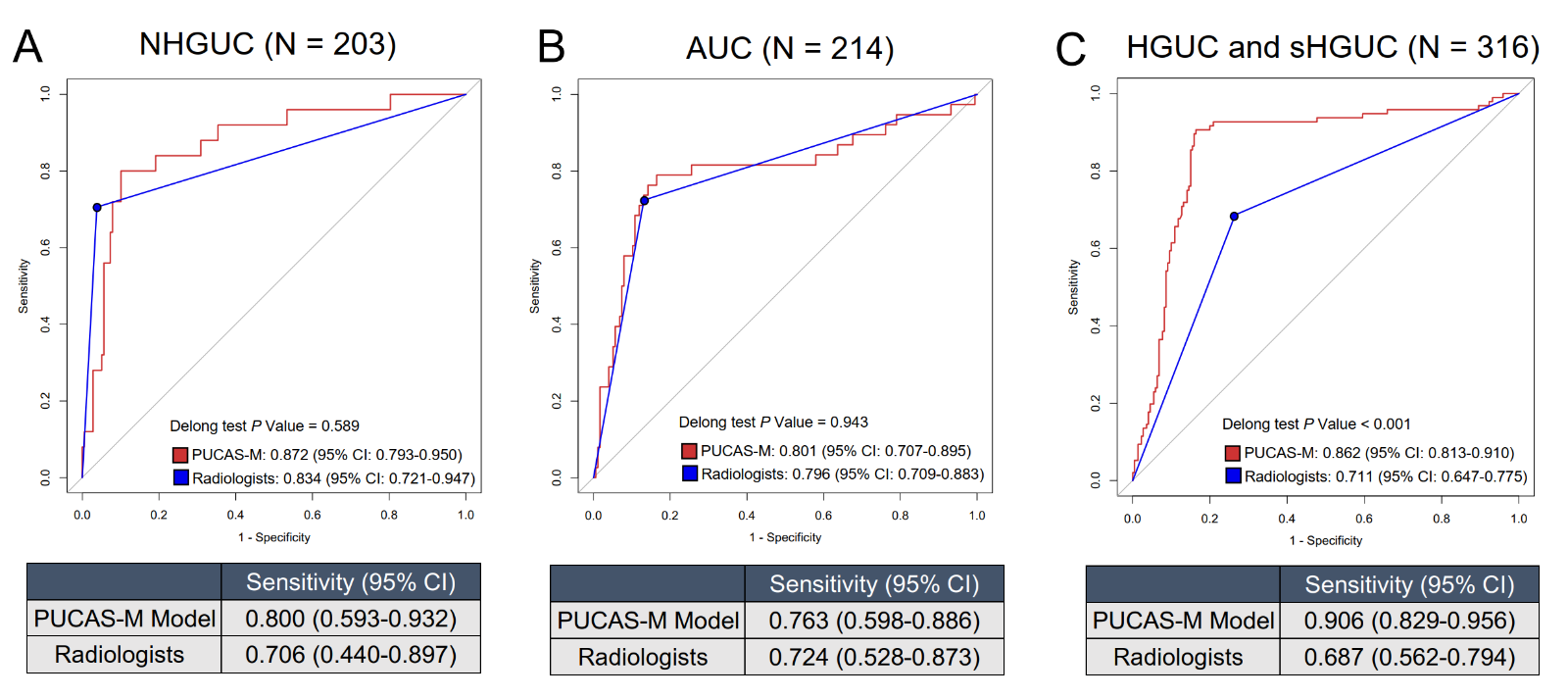
**Supplementary Figure 5. ROC curves and sensitivity of the PUCAS-M in different cytology results**

The above analyses were conducted in validation cohorts and their subgroups. (A) ROC curves and sensitivity of PUCAS-M in detecting MIUC in NHGUC subgroup of validation cohorts. (B) ROC curves and sensitivity of PUCAS-M in detecting MIUC in AUC subgroup of validation cohorts. (C) ROC curves and sensitivity of PUCAS-M in detecting MIUC in HGUC and sHGUC subgroup of validation cohorts. The *P* value of comparison between PUCAS-M and radiologist assessments was based on Delong test method.

ROC, Receiver Operating Characteristic Curve; PUCAS-M, The Precision Urine Cytology AI Solution for Muscle-invasion. CI, Confidence Interval. HGUC, High-Grade Urothelial Carcinoma; sHGUC, Suspicious for High-Grade Urothelial Carcinoma; AUC, Atypical Urothelial Cells; NHGUC, Negative for High-Grade Urothelial Carcinoma.

##
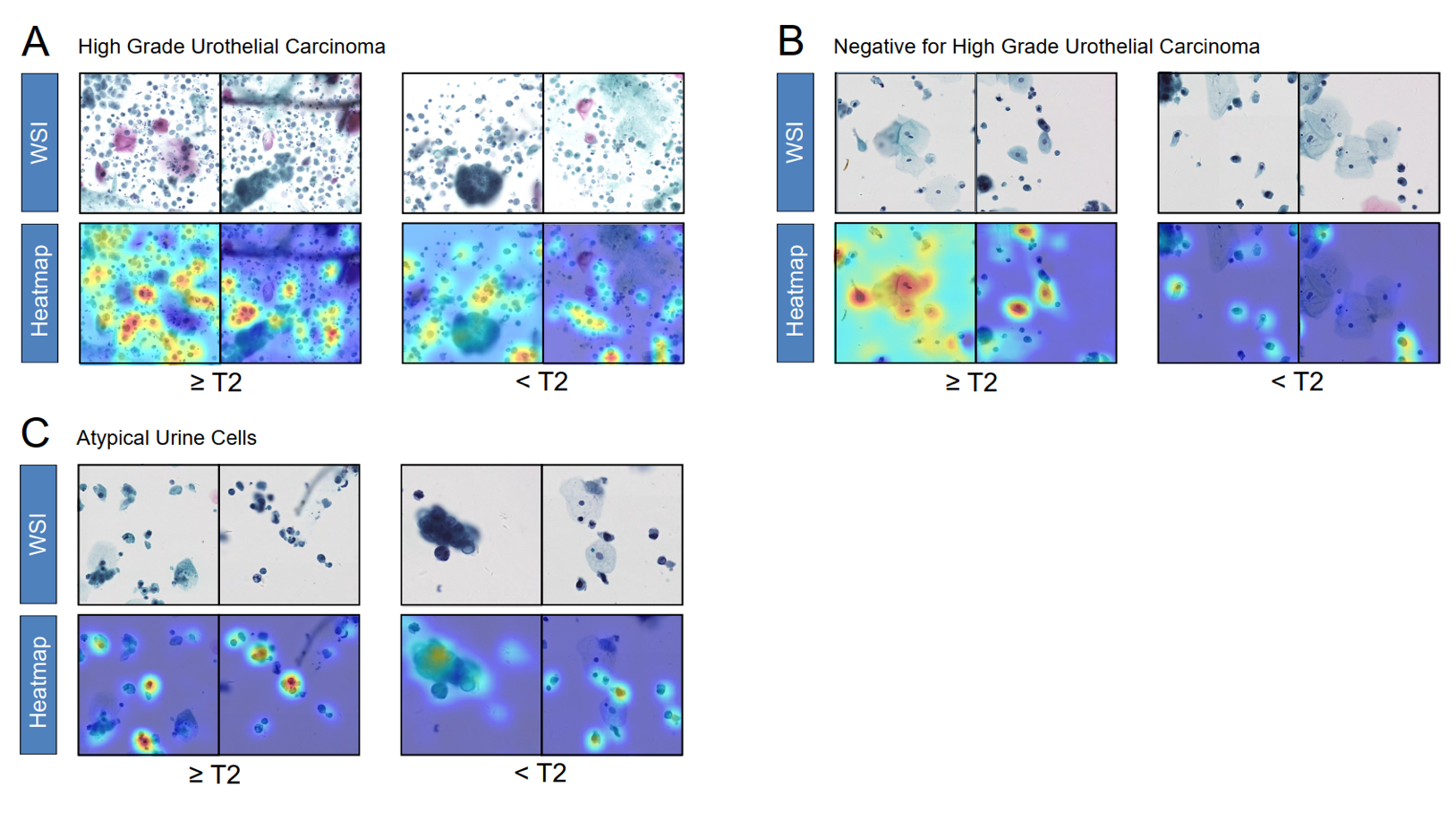
**Supplementary Figure 6. Examples of heatmaps of urine cytology WSIs in different MI status and cytology subgroups**

Examples of heatmaps of urine cytology WSIs in different MI status and cytology subgroups were generated to enhance the explainability of the model. The AI-identified high-risk regions (primarily localized to nuclei, cytoplasm, and peri-cellular background areas) demonstrated strong concordance with pathologist-annotated suspicious cells.

WSIs, Whole Slide Images. PUCAS-M, The Precision Urine Cytology AI Solution for Muscle-invasion. MIUC, Muscle Invasive Urothelial Carcinoma.

##
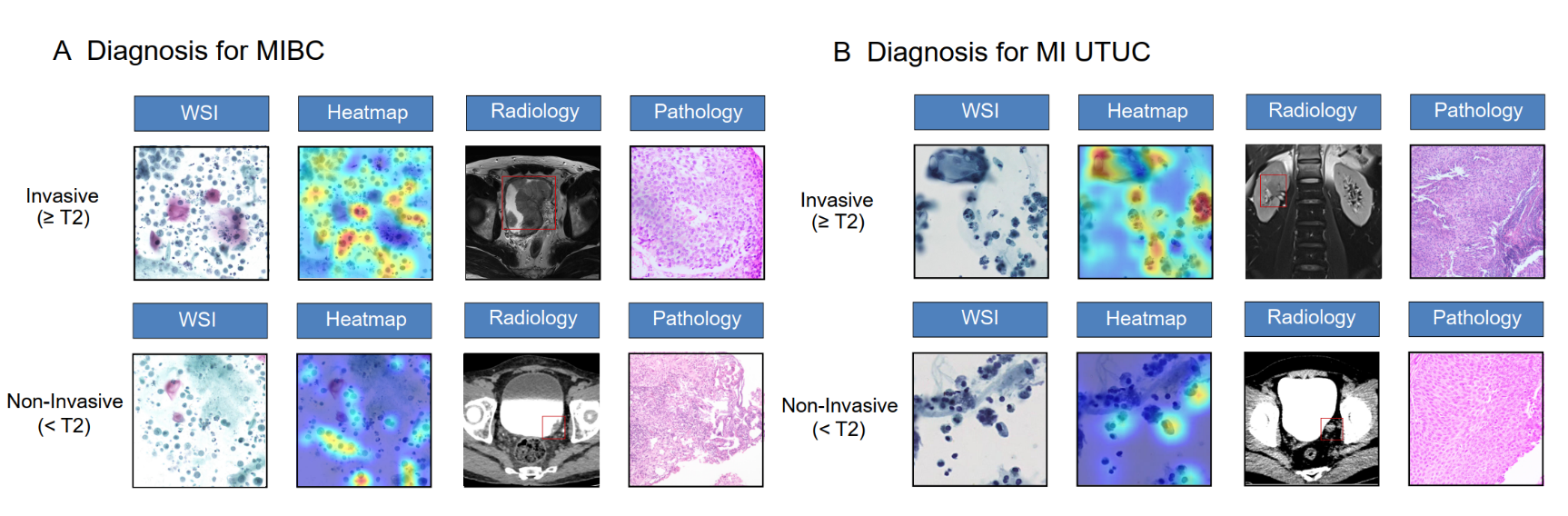
**Supplementary Figure 7. Examples of MIUC diagnoses by PUCAS-M in non-MI and MI, with corresponding radiology and pathology images**

Examples of heatmaps in different MI status and UC subgroups were generated to enhance the explainability of the model.

UC, Urothelial Carcinoma. PUCAS-M, The Precision Urine Cytology AI Solution for Muscle-invasion. MI, Muscle Invasion. MIBC, Muscle Invasive Bladder Cancer. UTUC, Upper-Tract Urothelial Carcinoma. WSI, Whole Slide Images.

##
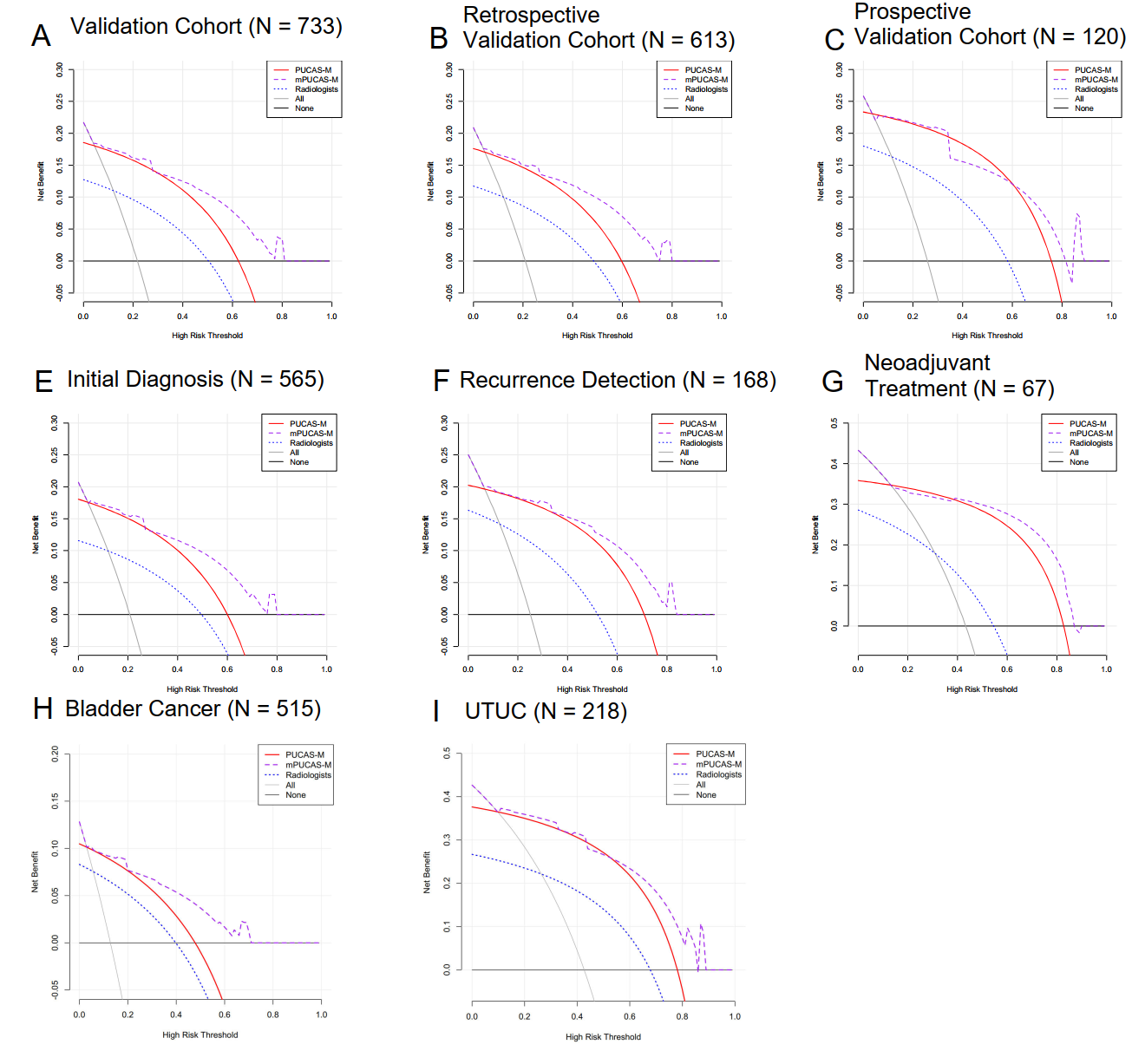
**Supplementary Figure 8. DCA curves of the mPUCAS-M in validation cohorts**

The above analyses were conducted in validation cohorts and their subgroups. X-axis indicates the threshold probability for pathology positive outcome and Y-axis indicates the net benefit. The purple line represents the net benefit when using the mPUCAS-M to detect MIUC. The red line represents the net benefit when using the PUCAS-M to detect MIUC. The thin solid line represents the assumption that all images are diagnosed as positive. While the thick solid line represents the assumption that all images are all diagnosed as negative. The results show that the mPUCAS-M can get clinical benefit in validation cohorts and subgroups.

DCA, Decision Clinical Analysis. PUCAS-M, The Precision Urine Cytology AI Solution for Muscle-invasion. mPUCAS-M, The Multi-modal Precision Urine Cytology AI Solution for Muscle-invasion; UTUC, upper-tract urothelial carcinoma.

## **Supplementary Figure 9. SHAP value of the mPUCAS-M**


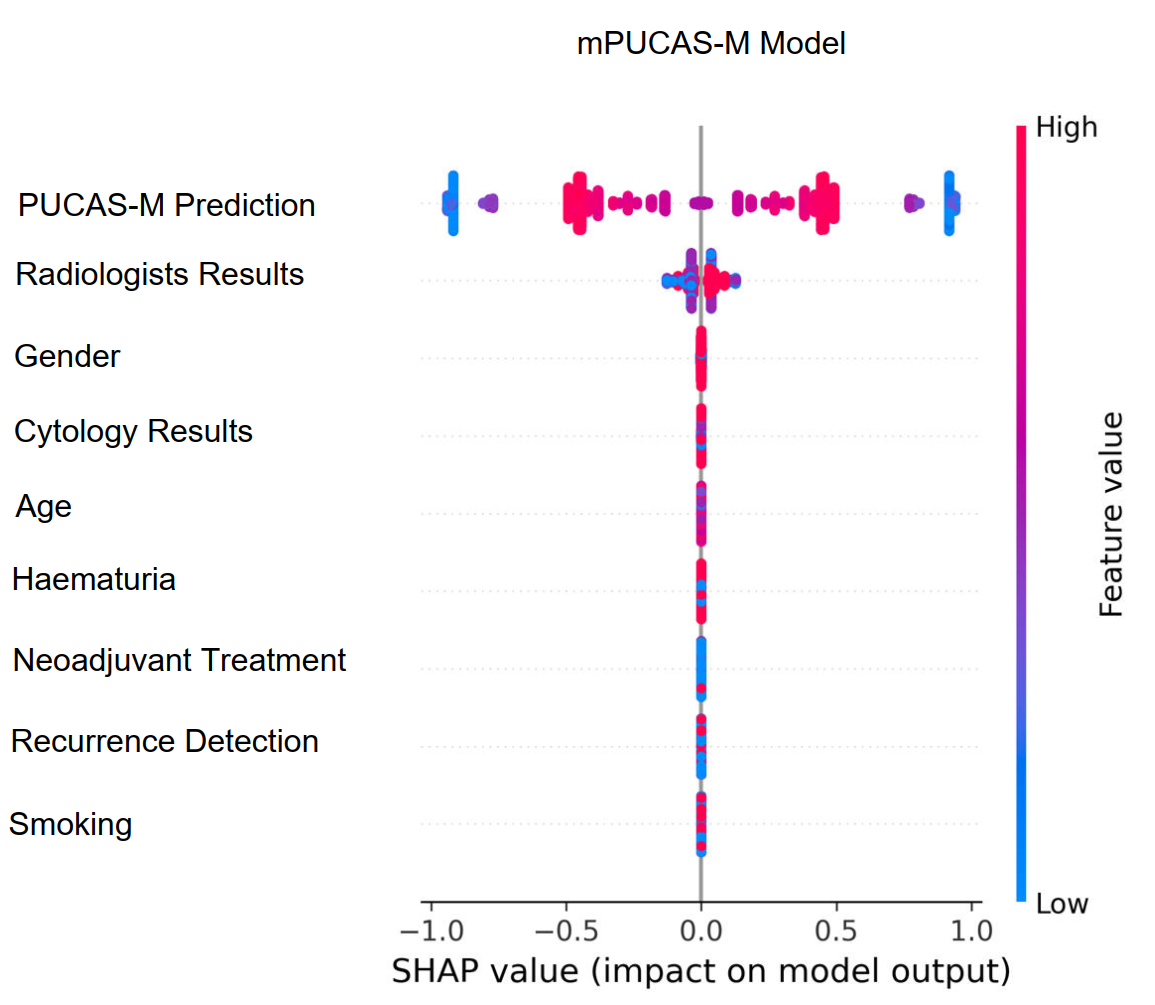


Shapley Additive exPlanations values illustrating feature importance in the mPUCAS-M model.

SHAP, Shapley Additive exPlanations. mPUCAS-M, The Multi-modal Precision Urine Cytology AI Solution for Muscle-invasion.

## **Supplementary Figure 10. mPUCAS-M to detect MIBC after neoadjuvant treatment**


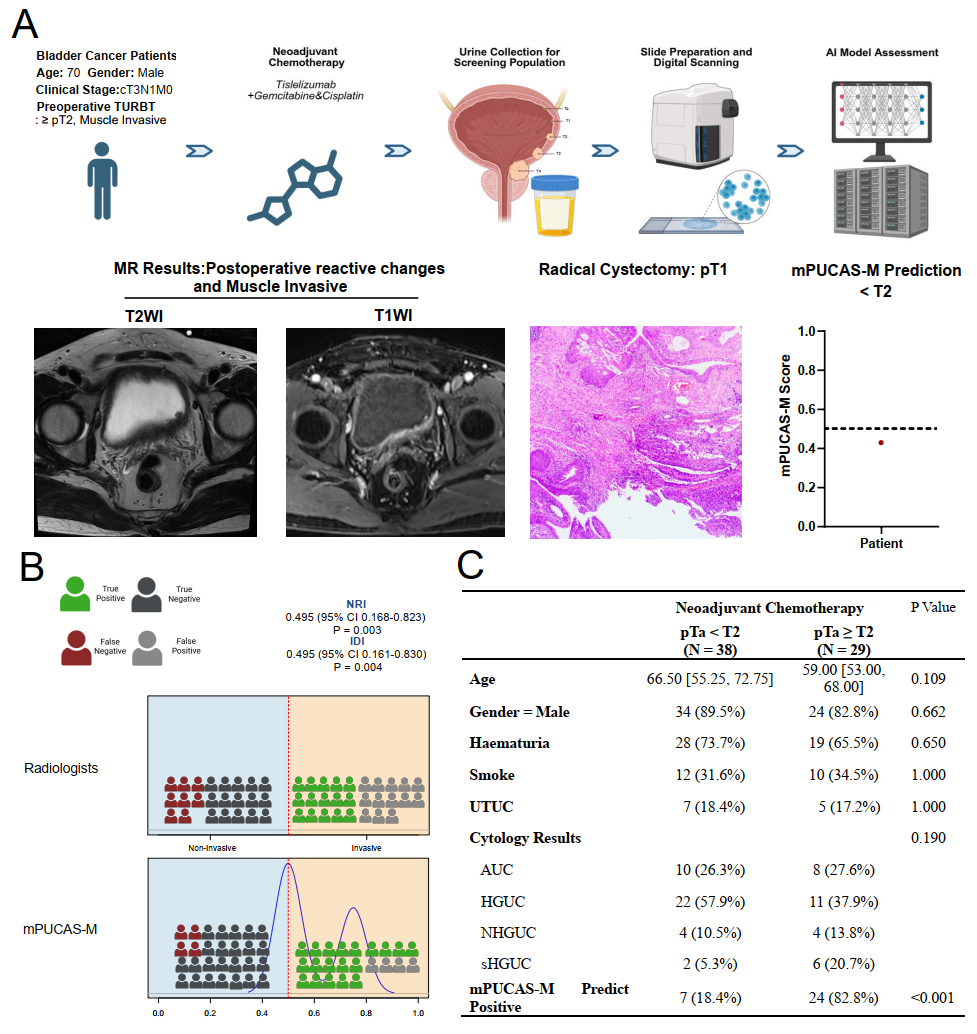


(A)Workflow of mPUCAS-M detecting MI status of MIBC patients after neoadjuvant treatment. It showed an example of mPUCAS-M in accurate identify pathology down stage in MIBC after neoadjuvant chemotherapy and immunotherapy. A cT3N1M0 MIBC patient (Male, age 70) received a planned treatment regimen consisting of four 21-day cycles of intravenous 200 mg tislelizumab on day 1, 1,000 mg/m² gemcitabine on days 1 and 8, and 70 mg/m² cisplatin on day 2. Before radical cystectomy surgery, MR was conducted and urine cytology was collected for mPUCAS-M analyses. And the MR results showed postoperative (TURBT) reactive changes and MI, but the postoperative (radical cystectomy) pathology showed pT1. mPUCAS-M presented a negative prediction result for T2, and is consistent with the final pathology. (B) Comparison of radiologist assessments and mPUCAS-M in detecting MI status of MIBC patients after neoadjuvant treatment. (C) Baseline characteristics of treatment responder and non-responder who received neoadjuvant treatment.

mPUCAS-M, The Multi-modal Precision Urine Cytology AI Solution for Muscle-invasion. MI, Muscle Invasion. MIBC, Muscle Invasive Bladder Cancer.

## **
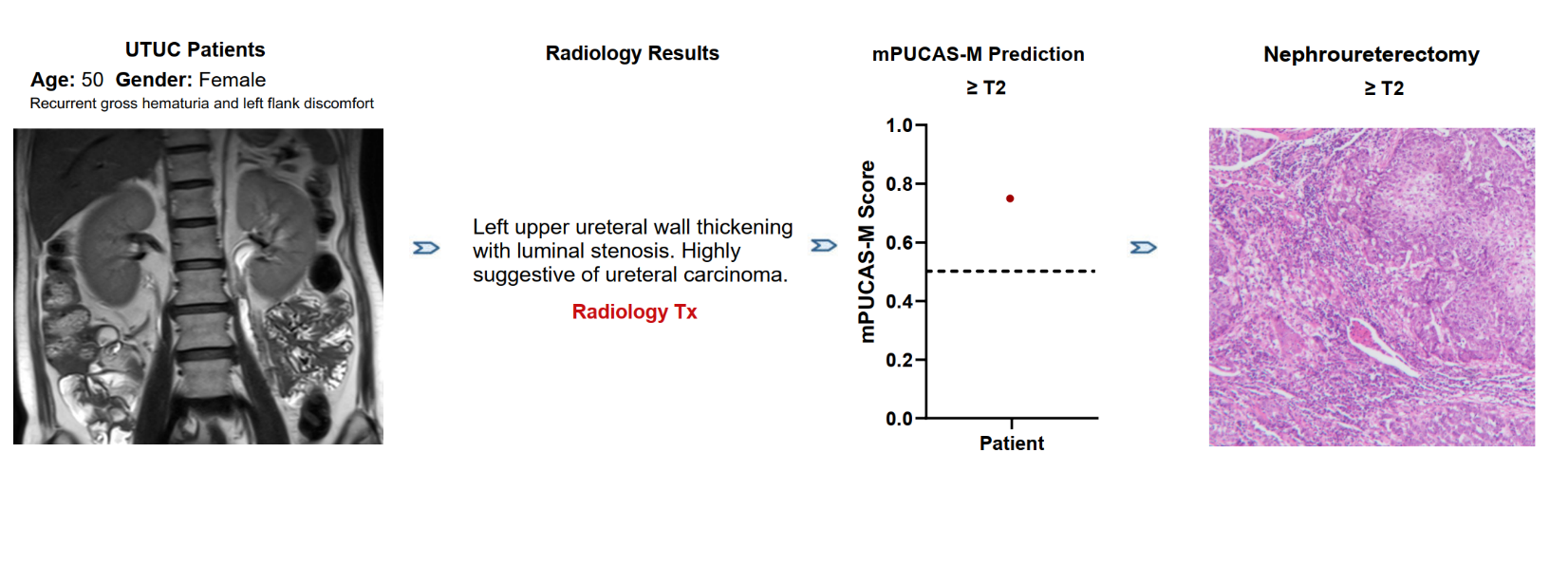
Supplementary Figure 11. One example of mPUCAS-M to detect hard-to-detect MI UTUC in initial diagnosis**

Corresponding MR, radiology results, mPUCAS-M prediction and pathology image of one hard-to-detect MI UTUC.

mPUCAS-M, The Multi-modal Precision Urine Cytology AI Solution for Muscle-invasion. MI, Muscle Invasive. UTUC, Upper-Tract Urothelial Carcinoma.

**References**

1. Ilse M, Tomczak J, Welling M. Attention-based deep multiple instance learning. International conference on machine learning; 2018: PMLR; 2018. p. 2127-36.

2. Wang C-Y, Bochkovskiy A, Liao H-YM. YOLOv7: Trainable bag-of-freebies sets new state-of-the-art for real-time object detectors. Proceedings of the IEEE/CVF Conference on Computer Vision and Pattern Recognition; 2023; 2023. p. 7464-75.

3. Liu Z, Mao H, Wu C-Y, Feichtenhofer C, Darrell T, Xie S. A convnet for the 2020s. Proceedings of the IEEE/CVF conference on computer vision and pattern recognition; 2022; 2022. p. 11976-86.

4. Radosavovic, Ilija , et al. "Designing Network Design Spaces." 2020 IEEE/CVF Conference on Computer Vision and Pattern Recognition (CVPR) IEEE, 2020.

5. Deng J, Dong W, Socher R, Li L-J, Li K, Fei-Fei L. Imagenet: A large-scale hierarchical image database. 2009 IEEE conference on computer vision and pattern recognition; 2009: Ieee; 2009. p. 248-55.

6. Luisier F, Blu T, Unser MJIToip. Image denoising in mixed Poisson–Gaussian noise. 2010; 20(3): 696-708.

7. Wei S, Qu Q, Zeng X, et al. Self-attention bi-lstm networks for radar signal modulation recognition. 2021; 69(11): 5160-72.

8. Kingma DP, Ba JJapa. Adam: A method for stochastic optimization. 2014.
